# Activation of the γ-Tubulin Complex by the Mto1/2 Complex

**DOI:** 10.1016/j.cub.2014.03.006

**Published:** 2014-04-14

**Authors:** Eric M. Lynch, Lynda M. Groocock, Weronika E. Borek, Kenneth E. Sawin

**Affiliations:** 1 Wellcome Trust Centre for Cell Biology, School of Biological Sciences, University of Edinburgh, Swann Building, Mayfield Road, Edinburgh EH9 3JR, UK

## Abstract

The multisubunit γ-tubulin complex (γ-TuC) is critical for microtubule nucleation in eukaryotic cells [1, 2], but it remains unclear how the γ-TuC becomes active specifically at microtubule-organizing centers (MTOCs) and not more broadly throughout the cytoplasm [3, 4]. In the fission yeast *Schizosaccharomyces pombe*, the proteins Mto1 and Mto2 form the Mto1/2 complex, which interacts with the γ-TuC and recruits it to several different types of cytoplasmic MTOC sites [5–10]. Here, we show that the Mto1/2 complex activates γ-TuC-dependent microtubule nucleation independently of localizing the γ-TuC. This was achieved through the construction of a “minimal” version of Mto1/2, Mto1/2[bonsai], that does not localize to any MTOC sites. By direct imaging of individual Mto1/2[bonsai] complexes nucleating single microtubules in vivo, we further determine the number and stoichiometry of Mto1, Mto2, and γ-TuC subunits Alp4 (GCP2) and Alp6 (GCP3) within active nucleation complexes. These results are consistent with active nucleation complexes containing ∼13 copies each of Mto1 and Mto2 per active complex and likely equimolar amounts of γ-tubulin. Additional experiments suggest that Mto1/2 multimers act to multimerize the fission yeast γ-tubulin small complex and that multimerization of Mto2 in particular may underlie assembly of active microtubule nucleation complexes.

## Highlights

• Free cytoplasmic “Mto1/2[bonsai]” complexes nucleate single microtubules in vivo
• Mto1/2[bonsai] puncta contain stoichiometric amounts of the γ-tubulin complex
• Mto1/2 multimers are required for assembly of γ-TuRC-like structures
• Mto2 multimers may drive formation of multimeric Mto1/2 complexes

## Teaser

Lynch et al. construct a “minimal” version of the fission yeast Mto1/2 complex that activates microtubule nucleation by the γ-tubulin complex independently of localization to canonical microtubule-organizing centers. Mto1/2 multimers appear to drive multimerization of the γ-tubulin small complex into active microtubule nucleation complexes.

## Results and Discussion

Mto1/2 localizes the γ-tubulin complex (γ-TuC) to all cytoplasmic microtubule-organizing centers (MTOCs) throughout the cell cycle [10, 11]. During interphase, Mto1/2 recruits the γ-TuC to the cytoplasmic face of the spindle pole body (SPB), to preexisting microtubules (MTs), and, less abundantly, to the nuclear envelope (NE), leading to MT nucleation from all of these sites. During anaphase elongation of the intranuclear mitotic spindle, Mto1/2 recruits the γ-TuC to the cytoplasmic face of the SPB to support astral MT nucleation, and during late mitosis, Mto1/2 recruits the γ-TuC to an equatorial MTOC (eMTOC) associated with the cytokinetic actomyosin ring, to generate a postanaphase MT array that stabilizes the actomyosin ring [12]. While Mto1/2 can be described as an attachment factor localizing the γ-TuC to different subcellular sites [3], *mto1Δ* cells exhibit a complete failure of de novo cytoplasmic MT nucleation [5, 13]. This suggests that Mto1/2 may be involved not only in γ-TuC localization but also somehow in γ-TuC activation. We envisaged two general models by which interaction of Mto1/2 with the γ-TuC could be important for generating active MTOCs. First, Mto1/2 might function only to localize the γ-TuC to prospective MTOC sites, and additional factors would then subsequently activate the γ-TuC at these sites. Alternatively, binding of Mto1/2 to the γ-TuC might simultaneously both localize and activate the γ-TuC [14]. We reasoned that if we could construct a mutant version of Mto1/2 that failed to localize to conventional MTOC sites but nevertheless promoted MT nucleation, this would provide strong support for the second model.

### Free Cytosolic Mto1/2[bonsai] Complexes Promote Spatially Random Microtubule Nucleation In Vivo

**Figure 1:**
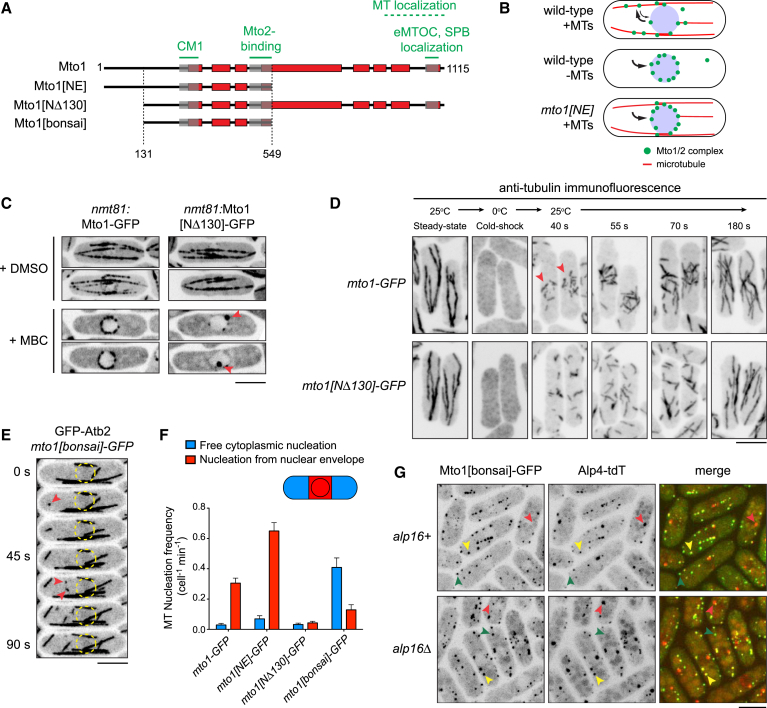
Mto1[bonsai], a “Minimal” Mto1 Truncation Mutant, Promotes Spatially Random Cytoplasmic Microtubule Nucleation (A) Schematic of full-length Mto1 and Mto1 truncation mutants. Dashed lines indicate N- and C-terminal truncation sites. Red boxes indicate predicted coiled coils. Gray boxes indicate regions involved in binding to γ-TuC (centrosomin motif 1; CM1), binding to Mto2, and localization to the equatorial microtubule-organizing center and spindle pole body (eMTOC and SPB). The region involved in binding to microtubules (MTs) is approximately defined [10]. (B) Cartoon summarizing the different means by which Mto1/2 complexes can become enriched on the nuclear envelope. (C) Localization of mildly overexpressed Mto1-GFP and Mto1[NΔ130]-GFP in control cells (+DMSO) and after MT depolymerization by methyl benzimidazol-2-yl-carbamate (MBC). Arrows indicate Mto1[NΔ130]-GFP at spindle pole bodies, but not more generally on the nuclear envelope (NE). (D) Anti-tubulin immunofluorescence showing time course and spatial distribution of MT regrowth after cold-induced MT depolymerization in *mto1-GFP* and *mto1[N*Δ*130]-GFP* cells. Arrows indicate regrowth from NE. (E) Time-lapse images (15 s interval) of GFP-tubulin in an *mto1[bonsai]-GFP* cell. Arrows indicate de novo MT nucleation events free in the cytoplasm. Circles indicate position of nucleus. Relative to GFP-tubulin, Mto1[bonsai]-GFP is too faint to be seen. See Movie S1 for corresponding movie. (F) Frequency of de novo MT nucleation (±SEM) free in the cytoplasm versus in the NE region in the cell genotypes indicated (see also Movie S1). The apparent low total MT nucleation frequency in *mto1-GFP* and *mto1[N*Δ*130]-GFP* cells is artifactual; in these cells, a significant proportion of nucleation occurs on preexisting MTs but cannot be quantified and thus is excluded from analysis (see Supplemental Experimental Procedures). (G) Localization of Mto1[bonsai]-GFP in wild-type (*alp16*+) and in *alp16Δ* cells, together with γ-TuC protein Alp4 (GCP2 homolog) fused to tandem-dimer Tomato (Alp4-tdT). Green arrows indicate Mto1[bonsai] puncta without Alp4. Yellow arrows indicate Mto1[bonsai] puncta with Alp4. Red arrows indicate spindle pole bodies (nucleoplasmic face), which contain Alp4 but not Mto1[bonsai]. Scale bars, 5 μm.

Because Mto1 C-terminal regions target the protein to MTs, the SPB, and the eMTOC [10], we first removed these regions by replacing the endogenous *mto1+* gene with GFP-tagged C-terminal truncations (Figure 1A; see also Supplemental Experimental Procedures available online). The smallest truncation mutant in which Mto1/2 remained functional was *mto1(1-549)-GFP* (Figure S1A). Unlike full-length Mto1-GFP, Mto1(1–549)-GFP localized almost exclusively to the NE, in the form of discrete puncta, and in these cells the γ-TuC protein Alp4 (GCP2 homolog) similarly localized to the NE (Figure S1B). We will therefore refer to Mto1(1–549) as “Mto1[NE].” In live-cell imaging experiments, both *mto1[NE]-GFP* and untagged *mto1[NE]* cells showed greater than 2-fold increased MT nucleation from the nuclear surface relative to wild-type cells (Figures 1F, S1E, and S1G; Movie S1), and Mto2-GFP in *mto1[NE]* cells also localized to the NE (Figure S1C). Interestingly, after drug- or cold-induced MT depolymerization, full-length Mto1 also redistributes to the NE ([13, 15]; see also Figures 1C and S2A). This suggests that normal steady-state localization of Mto1/2 may depend on a dynamic partitioning mechanism in which multiple prospective MTOC sites compete for a limited pool of Mto1/2 (Figure 1B). According to this view, in unperturbed wild-type cells, high-affinity/abundance sites such as MTs or the SPB largely outcompete the NE for recruitment of wild-type Mto1/2, but when MTs are absent (as in the case of full-length Mto1) or Mto1/2 cannot bind to such sites (as in the case of Mto1[NE]), competition is abolished, leading to increased Mto1/2 localization to the NE. Supporting this view of localization depending on competition, we found that both Mto1 and Mto2 are low-abundance proteins, each present at 1,200–1,400 molecules per cell (Figure S1D).

Truncation of the N terminus of full-length Mto1 (*mto1[NΔ130]*; Figure 1A) abrogated the redistribution to the NE that occurs upon MT depolymerization, both under conditions of normal expression and mild overexpression (Figures 1C and S2A). Consistent with this, MT regrowth after cold-induced depolymerization occurred randomly throughout the cytoplasm in *mto1[NΔ130]-GFP* cells, unlike wild-type cells, in which regrowth occurs mainly from the NE (Figures 1D and S2B; [15]). In live-cell imaging experiments, *mto1[NΔ130]-GFP* cells also showed strongly decreased MT nucleation in the vicinity of the NE compared to *mto1-GFP* cells (Figures 1F, S1F, and S1G).

Based on these results, we truncated *mto1* simultaneously at both N and C termini to make *mto1[131-549]-GFP*. Because this was found to be the smallest Mto1 fragment that supports MT nucleation in vivo (see below and Supplemental Experimental Procedures), we will refer to Mto1[131–549] as “Mto1[bonsai],” by analogy to other miniaturized but functional proteins [16, 17]. Strikingly, in live-cell imaging, *mto1[bonsai]-GFP* cells nucleated cytoplasmic MTs in a spatially random manner, with the majority of nucleation events occurring freely in the cytoplasm (Figures 1E, 1F, S1F, and S1G; Movie S1). Similar results were obtained in MT-regrowth experiments (Figure S2B). Interestingly, many freely nucleated microtubules quickly aligned and bundled with other MTs (Movies S1, S2, and S4). This led to steady-state distributions in which most MTs were oriented along the long axis of the cell in spite of spatially random nucleation (see for example Figures 3B and S2B–S2D).

**Figure 2:**
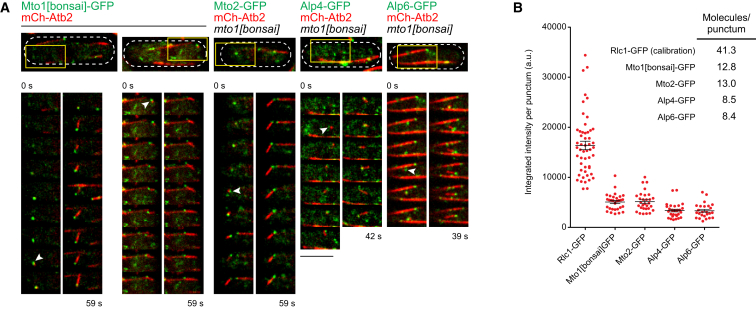
Direct Observation of Single Microtubule Nucleation by Mto1[bonsai] Puncta Reveals Protein Copy Number within Individual Puncta (A) Time-lapse images (3.3 s interval) of mCherry-microtubule nucleation (mCh-Atb2) from Mto1[bonsai]-GFP puncta as well as from Mto2-GFP, Alp4-GFP, and Alp6-GFP puncta in an untagged *mto1[bonsai]* background. Panels show single z sections or maximum projections of two adjacent z sections. Upper panels show entire cell area. Lower panels correspond to regions indicated by yellow boxes and show time-lapse images of nucleating puncta (white arrows). See Movie S2 for corresponding movies. (B) Fluorescent signal of individual Mto1[bonsai]-GFP, Mto2-GFP, Alp4-GFP, and Alp6-GFP puncta (±SEM), quantified at the time of microtubule nucleation. Calculation of molecules per punctum used Rlc1-GFP cytokinesis nodes as a fluorescence calibration standard [19]. Scale bar, 5 μm.

**Figure 3:**
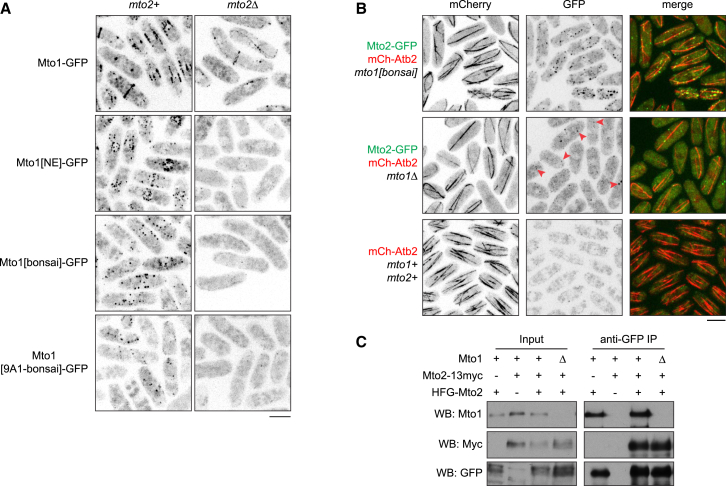
Assembly of Multimeric Puncta In Vivo (A) Images showing puncta of Mto1[NE]-GFP, Mto1[bonsai]-GFP, and Mto1[9A1-bonsai]-GFP in wild-type backgrounds (*mto2+*) and absence of puncta in *mto2*Δ backgrounds. Mto1[9A1-bonsai]-GFP puncta diffuse rapidly but are prominent in movies (see Movie S3). Full-length Mto1-GFP displays a weak but still detectable signal in *mto2Δ* backgrounds, due to its intrinsic ability to enrich at many MTOC sites ([9]; see also Figure 1A). (B) Mto2-GFP puncta together with mCherry-tubulin (mCh-Atb2) in *mto1[bonsai]* and *mto1*Δ cells. Arrows indicate examples of puncta in *mto1Δ* cells, which are relatively faint and seen more easily in movies or when Mto2-GFP is overexpressed (see Movie S5 and Figure S3E). Wild-type cells (*mto1+ mto2+*) provide a negative control for GFP fluorescence. (C) Mto2-13myc is coimmunoprecipitated with 6His-FLAG-GFP-Mto2 (HFG-Mto2), in both *mto1+* and *mto1*Δ backgrounds. Western blots were probed with antibodies against GFP, Myc, and Mto1. Scale bars, 5 μm.

Mto1[bonsai]-GFP itself was present throughout the cytoplasm as discrete, mobile puncta that did not localize to any conventional MTOC site (Figures 1G, 3A, S2C, and S2D; Movie S2). Most puncta also contained Alp4, although the relative signals of Mto1[bonsai] and Alp4 varied among puncta (Figure 1G). In many cells, multiple Mto1[bonsai]-GFP puncta appeared to be aligned, coincident with MT bundles (Figures 1G, S2C, and S2D), which suggested a possible association of Mto1[bonsai] with the MT lattice. However, in contrast to full-length Mto1-GFP, which does bind to the MT lattice [9, 10], three lines of evidence showed that Mto1[bonsai]-GFP localization to MT bundles is due not to lattice binding but rather to the presence of Mto1[bonsai]-GFP at minus ends of MTs that become bundled with other microtubules after nucleation (schematized in Figure S2E): First, while overexpressed full-length Mto1-GFP decorates MTs along their entire length [9, 10], overexpressed Mto1[bonsai]-GFP did not, remaining instead as discrete puncta (Figure S2C). A second piece of evidence came from introducing a nine-alanine (*9A1*) substitution mutation into the ∼60-amino-acid centrosomin motif 1 (CM1) region of *mto1* in *mto1[bonsai]-GFP* cells. The *9A1* mutation disrupts interaction with γ-TuC and abolishes Mto1-dependent MT nucleation in vivo, but it does not impair association of (full-length) Mto1[9A1]-GFP with the few cytoplasmic MTs that can appear in these cells via “escape” from the intranuclear mitotic spindle at the end of mitosis [13, 15, 18] (Figure S2D). Unlike Mto1[9A1]-GFP, Mto1[9A1-bonsai]-GFP puncta showed negligible localization to the few cytoplasmic MTs present in the cells (Figure S2D) and instead diffused freely throughout the cytoplasm (Movie S3). Therefore, the presence of Mto1[bonsai]-GFP in MT bundles depends on its ability to promote MT nucleation. Third, through live-cell imaging of Mto1[bonsai]-GFP together with mCherry-tubulin we were able to observe individual Mto1[bonsai]-GFP puncta nucleating single MTs (see below). In addition to demonstrating that Mto1[bonsai]-GFP is directly involved in nucleation, this explicitly showed Mto1[bonsai]-GFP puncta remaining bound to the minus ends of newly nucleated MTs during and after MT bundling (Figure 2A; Movie S2).

In live-cell imaging of *mto1[bonsai]-GFP* cells expressing mCherry-tubulin, all cytoplasmic MT nucleation events occurred from free Mto1[bonsai]-GFP puncta (30 puncta-associated events out of 30 total events). We also tagged Mto2, Alp4, and Alp6 (GCP3 homolog) with GFP in strains expressing untagged Mto1[bonsai], and in essentially all cases MT nucleation occurred from free puncta containing the GFP-tagged proteins (30 puncta-associated events out of 30 total events for Mto2-GFP, 30/32 for Alp4-GFP, and 25/26 for Alp6-GFP; see also Figure 2A; Movie S2). Immunoprecipitation experiments showed that Mto1[bonsai]-GFP physically interacts with the γ-TuC, and, as with Mto1-GFP, this requires both an intact Mto1 CM1 region and the presence of Mto2 (Figures S2F and S2G; [9, 13]). Taken together, our experiments with Mto1[bonsai] indicate that Mto1/2 can promote MT nucleation by the γ-TuC independently of localizing the γ-TuC to any conventional MTOC site—that is, localization and activation of the γ-TuC by Mto1/2 are separable, distinct activities.

### Microtubule Nucleation by Mto1/2[bonsai] Does Not Require γ-TuRC-Specific Proteins

In higher eukaryotes, the γ-TuC exists primarily as the γ-tubulin ring complex (γ-TuRC), which contains several copies of a heterotetrameric subcomplex (γ-tubulin small complex, γ-TuSC) arranged in a lock-washer-like structure [2–4, 20–22]. The γ-TuSC itself contains two copies of γ-tubulin and one copy each of GCP2 and GCP3, which are paralogs and highly conserved among eukaryotes [23, 24] (in fission yeast, Alp4 and Alp6, respectively). Assembly of γ-TuRC from multiple γ-TuSCs depends on additional “γ-TuRC-specific proteins” GCP4, GCP5, and GCP6, which are all predicted to be structurally similar to GCP2 and GCP3 [3, 25]. Fission yeast, unlike budding yeast, contains homologs of GCP4, GCP5, and GCP6 (in fission yeast, Gfh1, Mod21, and Alp16, respectively), and these physically associate with γ-TuSC proteins [6, 26, 27]. However, the precise mechanistic role of fission yeast γ-TuRC-specific proteins is uncertain, because simultaneous deletion of *gfh1+*, *mod21+*, and *alp16+* has only a modest effect on microtubule nucleation in vivo [27]. We thus investigated whether γ-TuRC-specific proteins, even if not essential in the context of Mto1/2 complexes containing full-length Mto1, might nevertheless be required to support the minimal Mto1/2[bonsai] complexes. We analyzed *alp16Δ mto1[bonsai]-GFP* cells, because Alp16 is required for association of both Gfh1 and Mod21 with γ-TuSC, and thus an *alp16Δ* single-deletion mutant phenocopies the *gfh1Δ mod21Δ alp16Δ* triple-deletion mutant [27, 28]. Mto1[bonsai]-GFP puncta in *alp16Δ* cells were indistinguishable from those in wild-type (*alp16*+) cells, and these puncta also contained Alp4 (Figure 1G). The frequency and distribution of MT nucleation in *mto1[bonsai]-GFP alp16Δ* cells was nearly identical to *mto1[bonsai]-GFP* (i.e., *alp16+*) cells, and this was also true for *mto1[NE]-GFP alp16Δ* versus *mto1[NE]-GFP* (*alp16+*) (Figure S1G; Movie S1). We conclude that γ-TuRC-specific proteins do not make a major contribution to Mto1/2[bonsai]-driven MT nucleation.

### Protein Copy Number within Individual Microtubule-Nucleation Complexes

The ability to image individual GFP-tagged Mto1/2[bonsai] and γ-TuC puncta as they nucleate single MTs allowed us to quantify protein copy number within individual nucleating puncta (Figures 2A and 2B). Using myosin light-chain Rlc1-GFP cytokinesis nodes as a fluorescence calibration standard [19], we measured signals of Mto1[bonsai]-GFP puncta and of Mto2-GFP, Alp4-GFP, and Alp6-GFP puncta in (untagged) *mto1[bonsai]* cells, just at the time of MT nucleation. Nucleating puncta contained an average of 12.8 and 13.0 molecules of Mto1[bonsai]-GFP and Mto2-GFP, respectively. These values are similar to the 13 protofilaments present in most template-nucleated MTs [29], and to the ∼13–14 γ-tubulin molecules thought to be present in a functional γ-TuRC [20, 30]. Nucleating puncta contained slightly more than half that number of molecules of Alp4-GFP and Alp6-GFP (average 8.5 and 8.4 molecules, respectively; Figure 2B). The near-equal values for Alp4 and Alp6 are consistent with puncta containing multiple copies of γ-TuSC, with numbers similar to those expected for GCP2 and GCP3 in a γ-TuRC [20, 30, 31]. Overall, our results suggest that the puncta we observe nucleating single MTs in vivo correspond to single macromolecular complexes with properties similar to single γ-TuRCs. The stoichiometry of Mto1[bonsai]:Mto2:Alp4:Alp6 in these γ-TuRC-like complexes is approximately 2:2:1:1, suggesting that each γ-TuSC in an actively nucleating complex may be associated with two copies each of Mto1[bonsai] and Mto2.

### Role of Mto1 and Mto2 in Assembly of Multimeric Nucleation Complexes

Because nucleating puncta contain multiple copies of both γ-TuSC and Mto1/2 proteins, we next investigated how puncta assemble. Does a multimeric Mto1/2 complex drive assembly of a multimeric γ-TuC, or vice versa? Previous work has shown that in *mto1Δ* cells, γ-TuSC proteins are not observed as freely diffusing cytoplasmic puncta [28], suggesting that Mto1/2 is required for multimerization of free γ-TuSCs. As mentioned above, we found that Mto1[9A1-bonsai]-GFP is present in puncta even though it cannot bind the γ-TuC (Figures 3A, S2D, S2F, and S2G; Movie S3). Mto1[9A1-bonsai]-GFP puncta often appeared less numerous than Mto1[bonsai]-GFP puncta (Figures 3A and S2D), but because of their rapid diffusion in the cytoplasm (Movie S3), this was difficult to determine definitively. We therefore introduced the *9A1* mutation into Mto1[NE]-GFP. Mto1[9A1-NE]-GFP localized as stable NE-associated puncta that, like Mto1[9A1-bonsai]-GFP puncta, did not promote microtubule nucleation (Figure S3B). These results strongly suggest that Mto1/2 multimerization (i.e., puncta formation) can occur independently of interaction with γ-TuC and thus may drive assembly of multimeric γ-TuC. We note that it remains possible that further interaction with the γ-TuC could help to cooperatively stabilize multimeric Mto1/2 complexes [32] or regulate protein copy number within complexes.

How does Mto1/2 multimerize? In *mto2*Δ cells, full-length Mto1-GFP localizes to conventional MTOCs, but the Mto1-GFP signal is less intense than in wild-type cells (Figure 3A; [9]). In immunoprecipitation experiments we found that Mto1 and Mto2 are the only major stoichiometric components of Mto1/2 complex isolated from cells (Figure S3A). This led us to hypothesize that Mto2 may be involved in multimerizing Mto1. Consistent with this, when *mto2+* was deleted in *Mto1[NE]-GFP* and *Mto1[bonsai]-GFP* cells, puncta were completely absent and no de novo cytoplasmic MT nucleation was observed (Figure 3A; Movie S4). Puncta were also absent from *Mto1[9A1-bonsai]-GFP mto2Δ* cells (Figure 3A; Movie S3) and from *mto1(1-500)-GFP* (*mto2+*) cells, in which Mto2 is present but the Mto2-binding region of Mto1 (Mto1 amino acids 461–549) is disrupted (Figure S3C; [9]). We also found that free cytoplasmic Alp4 puncta were absent in *mto2Δ mto1[bonsai]-GFP* cells, consistent with γ-TuC multimerization depending on the presence of Mto1/2 multimers (Figure S3D).

By contrast, when we imaged Mto2-GFP in *mto1Δ* cells, we observed free cytoplasmic Mto2-GFP puncta (Figure 3B). Although these puncta were faint in still images, they could clearly be seen diffusing very rapidly by time-lapse imaging (Movie S5). They were also considerably more apparent when Mto2-GFP was mildly (∼5× to 10×) overexpressed in *mto1*Δ cells, whereas similarly overexpressed Mto1[bonsai]-GFP in *mto2*Δ cells failed to form puncta and did not promote MT nucleation (Figures S3E and S3F). Overall, this suggests that multimeric Mto2 can exist in the absence of Mto1. Consistent with this, we found that Mto2 can be coimmunoprecipitated with itself, not only from wild-type but also from *mto1Δ* cell extracts (Figure 3C).

**Figure 4:**
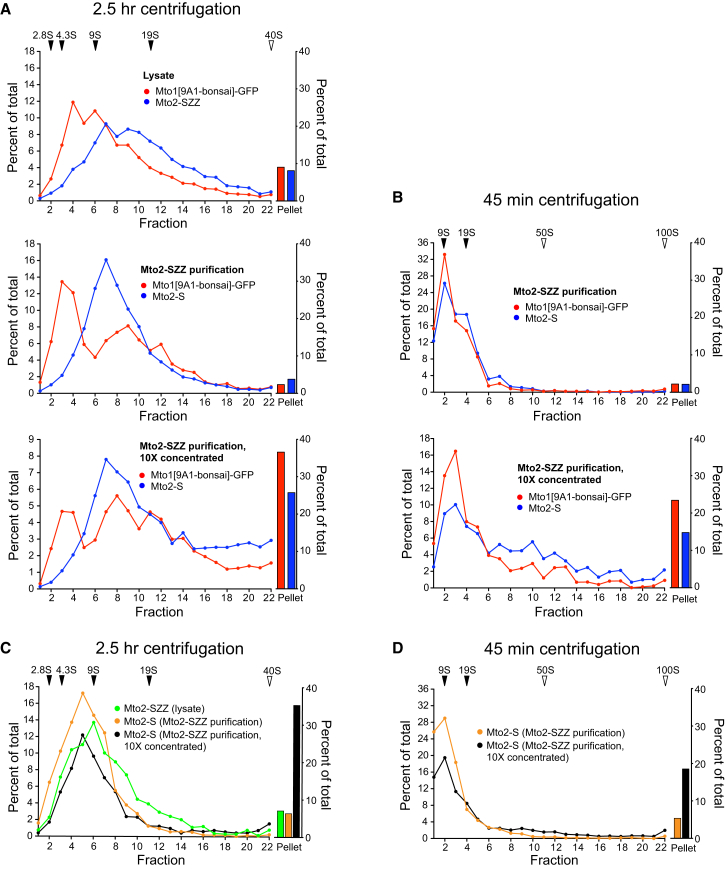
Concentration-Dependent Assembly of Mto1/2[bonsai] and Mto2 into Higher-Order Complexes In Vitro (A) Sedimentation of Mto1[9A1-bonsai]-GFP and Mto2-SZZ (or Mto2-S) in cell lysates, after Mto2-based partial purification from lysates, and after concentration of the partially purified material, as indicated. Quantitative western blotting from 2.5 hr isokinetic glycerol density-gradient centrifugation is shown. Solid triangles indicate measured S values; open triangles indicate extrapolated S values (see Figure S3H). (B) Samples as in (A), but centrifuged for only 45 min. (C) Sedimentation of Mto2-SZZ in *mto1Δ* cell lysates, Mto2-S after partial purification from *mto1Δ* lysates, and Mto2-S after concentration of the partially purified material from *mto1Δ* lysates. Centrifugation and quantification were as in (A). (D) Samples as in (C), but centrifuged for 45 min.

To further investigate γ-TuC-independent multimerization of Mto1/2, we analyzed endogenous Mto1/2[9A1-bonsai]-GFP complex by glycerol density-gradient centrifugation and quantitative western blotting (Figure 4). In these experiments, Mto2 was tagged with SZZ to enable partial purification by binding to immunoglobulin G beads and cleavage with tobacco etch virus (TEV) protease (see Supplemental Experimental Procedures); the SZZ tag did not impair Mto2 function (Figure S3G). In cell lysates, Mto2-SZZ sedimented as a very broad peak centered at ∼14S, with a full width at half maximum (FWHM) of eight or nine fractions (Figure 4A). By contrast, FWHM for monodisperse size standards was two or three fractions (Figure S3H). Sedimentation of Mto1[9A1-bonsai]-GFP was also broad but centered at ∼8S, indicating partial dissociation from Mto2-SZZ. Overall, this suggests that Mto1/2[9A1-bonsai]-GFP in cell lysates is likely present in several multimeric states.

Following partial purification, Mto2-S (the TEV protease cleavage product of Mto2-SZZ) was enriched 3,400-fold relative to total protein (Supplemental Experimental Procedures) and again sedimented as a broad peak (FWHM ∼ five fractions), centered at ∼12S (Figure 4A). Copurifying Mto1[9A1-bonsai]-GFP showed a more complex bimodal profile, which likely reflects further dissociation from Mto2-S after partial purification. Interestingly, after 10-fold concentration, both Mto2-S and Mto1[9A1-bonsai]-GFP sedimented as much larger species, represented by a long “tail” in the lower half of the gradient and a significantly increased proportion in the pellet fraction (corresponding to complexes > 40S). To examine larger species in more detail, we repeated this experiment, but with a shorter centrifugation time (Figure 4B). This resolved the earlier “>40S pellet” fraction into a range of larger species, with 15%–20% of total Mto2-S and Mto1[9A1-bonsai]-GFP still appearing in the pellet (>100S) after the shorter centrifugation.

These observations are consistent with a concentration-dependent higher-order multimerization of the Mto1/2 complex, possibly in the form of a polymer with no intrinsic fixed length [22]. Importantly, although Mto2-S was enriched 3,400-fold after partial purification, Mto1[9A1-bonsai]-GFP was enriched only 200-fold, and γ-tubulin was not enriched at all (Supplemental Experimental Procedures). This suggests that Mto1[9A1-bonsai]-GFP may make only a minor contribution to the assembly of higher-order complexes observed here and, moreover, that this assembly does not depend on any residual weak interaction with the γ-TuC.

To extend these observations, we repeated the above experiments in an *mto1*Δ background, both in cell lysates and after partial purification of Mto2-S (Figures 4C and 4D). In the absence of Mto1, Mto2-SZZ in cell lysates sedimented as a broad peak centered at 9S (FWHM ∼ six fractions), consistent with the presence of Mto2 multimers. The profile of partially purified Mto2-S was only modestly different (peak center 8S, FWHM ∼ five fractions). However, after 10-fold concentration, we observed a greatly increased proportion of Mto2-S in the pellet, with both long and short centrifugation times (Figures 4C and 4D). Interestingly, the ∼40–100S “tail” of concentrated Mto2-S species obtained from the *mto1[9A1-bonsai]-GFP* background (Figure 4B) was absent in material from *mto1*Δ cells (Figure 4D), which could suggest that Mto1[9A1-bonsai]-GFP contributes to the cooperative assembly of higher S-value complexes. However, at the same time, because a greater proportion of Mto2-S was observed in the pellet from *mto1*Δ cells compared to *mto1[9A1-bonsai]-GFP* cells, this could equally suggest that Mto1[9A1-bonsai]-GFP actually limits the extent of Mto2-S assembly into very large (>100S) complexes. Taken together, our results indicate that although Mto1 may influence the assembly of Mto1/2 complexes, Mto1 is not absolutely necessary for Mto2 to assemble in higher-order complexes.

### Conclusions

Overall, our experiments with Mto1/2[bonsai], a “minimal” Mto1/2 complex, demonstrate that Mto1/2 complex can activate MT nucleation by the fission yeast γ-TuC independently of localizing the γ-TuC to any specific location within the cell. While γ-TuRC-specific proteins are not required, multimeric Mto1/2 puncta are critical for MT nucleation and can form independently of interaction with the γ-TuC. Our results suggest that multimerization of γ-TuSCs by Mto1/2 may generate the same type of supramolecular architecture as is found in conventional γ-TuRCs, but via an alternative mode of assembly (Figure S3I). It will be interesting to investigate where such alternative γ-TuRC-like complexes may be used in other eukaryotic systems, including metazoans, as mutations in a mammalian homolog of Mto1, CDK5RAP2, lead to microcephaly [33, 34]. Combining localization and multimerization functions in wild-type Mto1/2 may be a particularly efficient method of spatially controlling MT nucleation, especially when the total number of MTs (and nucleation complexes) in the cell is low and when different prospective MTOC sites compete for these complexes.

Single-molecule electron microscopy studies have suggested that conformational changes within interacting γ-TuSCs may be important in generating an active MT nucleator [3, 22, 35]. This idea is attractive but still awaits experimental confirmation. The CM1 region of Mto1 is conserved in proteins from yeast to humans and is required for interaction with the γ-TuC [13, 36, 37], and there is good evidence that the CDK5RAP2 CM1 region alone can function as an activator of the mammalian γ-TuRC, although the mechanism remains unclear [14]. Our results do not address whether the Mto1 CM1 region alone has a similar specific activating role, because in the fission yeast system, as we have shown here, MT nucleation by Mto1[bonsai] further requires Mto2-dependent multimerization. In the future, such questions may best be addressed through reconstitution of complete MT-nucleating complexes from purified components.

## Experimental Procedures

Standard yeast genetic methods [38, 39] were used throughout. All fluorescence microscopy used a spinning-disk fluorescence microscope, as described previously [28, 40], with cells mounted on medium agarose pads between a slide and coverslip [41]. Further details of these and biochemistry experiments can be found in Supplemental Experimental Procedures.

## Supplemental Information

Document S1. Figures S1–S3 and Supplemental Experimental Procedures

Movie S1. *mto1[NE]-GFP* cells Exhibit Increased Microtubule Nucleation from the Nuclear Envelope, While *mto1[bonsai]-GFP* Cells Exhibit Spatially Random Microtubule Nucleation GFP-tubulin (GFP-Atb2) in *mto1-GFP*, *mto1[NE]-GFP*, and *mto1[bonsai]-GFP* cells, in both *alp16+* (wild-type) and *alp16*Δ backgrounds. In *alp16Δ* cells, none of the “γ-TuRC-specific” proteins Gfh1 (GCP4 homolog), Mod21 (GCP5), or Alp16 (GCP6) are associated with the γ-tubulin small complex (γ-TuSC). Movies of *alp16+* cells correspond to the images shown in Figures 1E and S1E. GFP-tagged Mto1 is too faint to be seen here relative to GFP-tubulin. All sequences play twice, with asterisks indicating nucleation events during the first run. z series were acquired every 5 s. Maximum projections of nine z sections are shown. Movie plays at 15 frames per second (fps).

Movie S2. Microtubules Are Nucleated Directly from Puncta Containing Mto1[bonsai]-GFP, Mto2-GFP, Alp4-GFP, and Alp6-GFP mCherry-microtubule (mCh-Atb2) nucleation from Mto1[bonsai]-GFP puncta, and from Mto2-GFP, Alp4-GFP, and Alp6-GFP puncta in an (untagged) *mto1[bonsai]* background. Movies correspond to images shown in Figure 2A. In the lower Mto1[bonsai]-GFP movie, an Mto1[bonsai]-GFP punctum nucleates an mCherry-microtubule that is subsequently incorporated into a microtubule bundle. Each sequence plays twice, with a box indicating the relevant punctum during the first run. z series were acquired every 1.63 s. Movies show single z sections or maximum projections of two adjacent z sections. Movie plays at 15 fps.

Movie S3. Formation of Mto1 Puncta Requires Mto2 Mto1[9A1-bonsai]-GFP in *mto2+* (wild-type) and *mto2*Δ cells. Mto1[9A1-bonsai] does not interact with the γ-tubulin complex (Figure S2) and thus allows investigation of Mto1/2 complex organization independently of its association with the γ-tubulin complex. Related examples of other Mto1 variants are shown in Figure 3A. z series were acquired every 4.5 s. Maximum projections of nine z sections are shown. Movie plays at 15 fps.

Movie S4. *mto1[NE]-GFP* and *mto1[bonsai]-GFP* Cells Are Completely Defective in Cytoplasmic Microtubule Nucleation in the Absence of Mto2 GFP-tubulin (GFP-Atb2) in *mto1+* (wild-type), *mto1[NE]-GFP*, and *mto1[bonsai]-GFP* cells, in both *mto2+* (wild-type) and *mto2*Δ backgrounds. GFP-tagged Mto1 is too faint to be seen here relative to GFP-tubulin. In *mto1+ mto2Δ* cells, cytoplasmic microtubules are nucleated from the spindle pole body only, during both mitosis (astral microtubules from mitotic spindle, right-most cell in bottom-left panel) and interphase (arrow in bottom-left panel). By contrast, in *mto1[NE]-GFP mto2Δ* and *mto1[bonsai]-GFP mto2Δ* cells, cytoplasmic microtubules are never nucleated de novo (0 events in 50 cells imaged over 500 s; data not shown). Indeed, when cytoplasmic microtubules are present in these cells, they are derived from intranuclear mitotic spindle microtubules that escape into the cytoplasm at the end of mitosis. If these cytoplasmic microtubules depolymerize completely, they never return, leading to many cells without any cytoplasmic microtubules. z series were acquired every 5 s. Max projections of nine z sections are shown. Movie plays at 15 fps.

Movie S5. Formation of Mto2 Puncta Does Not Require Mto1 Mto2-GFP and mCherry-tubulin (mCh-Atb2) in *mto1[bonsai]* and *mto1*Δ cells. mCh-Atb2 is also shown in *mto1+ mto2+* cells (wild-type, without any GFP-tagged protein) as a negative control for GFP fluorescence. Movies correspond to images shown in Figure 3B. When cytoplasmic microtubules are present in *mto1Δ* cells, they are always derived from intranuclear mitotic spindle microtubules that escape into the cytoplasm. z series were acquired every 1.63 s for 40 time points. Maximum projections of eight z sections are shown. Movie plays at 15 fps.

Document S2. Article plus Supplemental Information
