# Supplementary material for: Activation of the γ-Tubulin Complex by the Mto1/2 Complex
Source: Curr Biol. 2014 Apr 14;24(8):896–903. doi: 10.1016/j.cub.2014.03.006 (PMC3989768; doi:10.1016/j.cub.2014.03.006)
Supplement: Document S1. Figures S1–S3 and Supplemental Experimental Procedures [file mmc1.pdf]

Current Biology, Volume *24*

Supplemental Information

Activation of the γ-Tubulin Complex by the Mto1/2 Complex

Eric M. Lynch, Lynda M. Groocock, Weronika E. Borek, and Kenneth E. Sawin

Suppl. Fig. S1

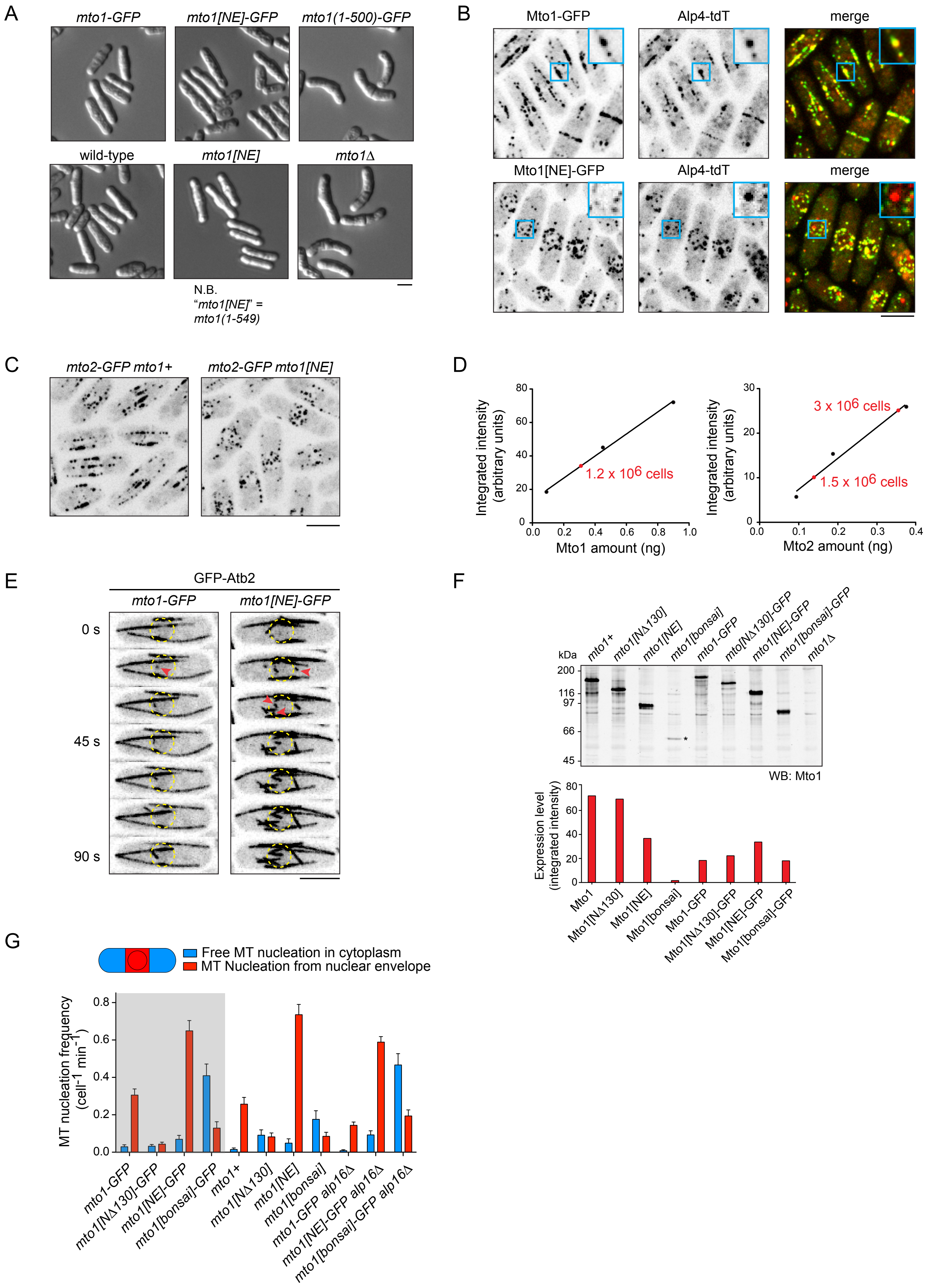
A
mto1-GFP
mto1[NE]-GFP
mto1(1-500)-GFP
wild-type
mto1[NE]
mto1Δ
N.B.
"mto1[NE]" =
mto1(1-549)
B
Mto1-GFP
Alp4-tdT
merge
Mto1[NE]-GFP
Alp4-tdT
merge
C
mto2-GFP mto1+
mto2-GFP mto1[NE]
D
Integrated intensity (arbitrary units)
1.2 x 10^6 cells
Mto1 amount (ng)
3 x 10^6 cells
1.5 x 10^6 cells
Mto2 amount (ng)
E
GFP-Atb2
mto1-GFP
mto1[NE]-GFP
0 s
45 s
90 s
F
kDa
mto1+
mto1[NΔ130]
mto1[NE]
mto1[bonsai]
mto1-GFP
mto[NΔ130]-GFP
mto1[NE]-GFP
mto1[bonsai]-GFP
mto1Δ
200
116
97
66
45
WB: Mto1
Expression level (integrated intensity)
Mto1
Mto1[NΔ130]
Mto1[NE]
Mto1[bonsai]
Mto1-GFP
Mto1[NΔ130]-GFP
Mto1[NE]-GFP
Mto1[bonsai]-GFP
G
Free MT nucleation in cytoplasm
MT Nucleation from nuclear envelope
MT nucleation frequency (cell^-1 min^-1)
mto1-GFP
mto1[NΔ130]-GFP
mto1[NE]-GFP
mto1[bonsai]-GFP
mto1+
mto1[NΔ130]
mto1[NE]
mto1[bonsai]
mto1-GFP alp16Δ
mto1[NE]-GFP alp16Δ
mto1[bonsai]-GFP alp16Δ

Supplemental Figure S1: Localization of Mto1[NE], protein levels, and microtubule nucleation frequencies.

(A) Cell morphology for the indicated strains after return-to-growth from stationary phase [S1, S2].

(B) Localization of full-length Mto1-GFP or Mto1[NE]-GFP, together with the γ-TuC protein Alp4 (GCP2 homolog) fused to tandem-dimer Tomato (Alp4-tdT). Large panels are maximum projections of the entire cell volume. Insets correspond to the blue squares in large panels and are maximum projections of the three Z-sections containing the spindle pole body (SPB). Alp4-tdT colocalizes with Mto1[NE]-GFP, except at the SPB itself, where Mto1[NE]-GFP is absent but Alp4-tdT is present, presumably because of its association with the Mto1 paralog Pcp1 on the intranuclear face of the SPB [S3, S4].

(C) Localization of Mto2-GFP in *mto1+* and *mto1[NE]* cells.

(D) Amounts of Mto1 and Mto2 in fission yeast extracts from the indicated numbers of cells (red data points), determined by quantitative western blotting and calibration with known amounts of recombinant Mto1 and Mto2 (black data points). These correspond to ~1200-1400 molecules per cell for both Mto1 and Mto2.

(E) Time-lapse images (15 s interval) of GFP-tubulin (GFP-Atb2) in *mto1-GFP* and *mto1[NE]-GFP* cells. Arrows indicate *de novo* microtubule (MT) nucleation events. Circles indicate position of the nucleus. Relative to GFP-tubulin, Mto1-GFP and Mto1[NE]-GFP are too faint to be seen here. See Suppl. Movie S1 for corresponding movies.

(F) Anti-Mto1 Western blot showing Mto1 levels for the indicated strains. Asterisk indicates untagged Mto1[bonsai]. Corresponding quantification of expression levels is shown below.

(G) Frequency of *de novo* MT nucleation (±SEM) free in the cytoplasm vs. in the nuclear envelope region for the indicated strains, as determined from time-lapse imaging of GFP-tubulin. Gray box shows data reproduced from Fig. 1F, for comparison. The apparent low total MT nucleation frequency in full-length *mto1* and *mto1[NΔ130]* strains, both in the presence or absence of a GFP tag and/or *alp16*Δ, is an artifact of measurement methods; in these cells, a significant proportion of nucleation occurs on pre-existing MTs but cannot be quantified and thus is excluded from analysis. Taking this into consideration, appropriate comparisons can be made between different strains. The lower nucleation frequency in untagged *mto1[bonsai]* cells compared to *mto1[bonsai]-GFP* cells is likely due to the lower steady-state levels of untagged Mto1[bonsai] protein, as shown in panel (F). Bars, 5 µm.

Suppl. Fig. S2

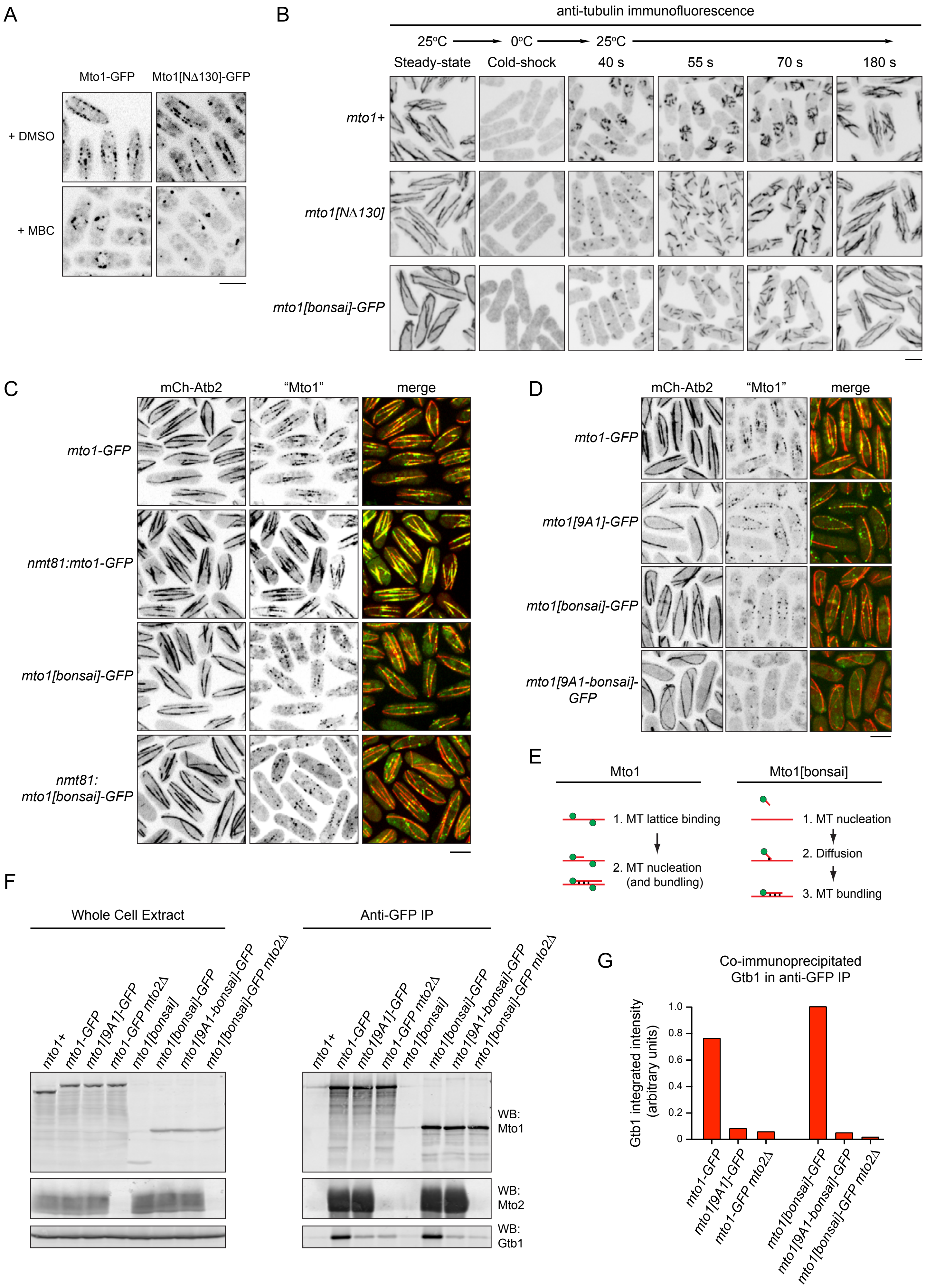
A
Mto1-GFP
Mto1[NΔ130]-GFP
+ DMSO
+ MBC
B
anti-tubulin immunofluorescence
25°C
0°C
25°C
Steady-state
Cold-shock
40 s
55 s
70 s
180 s
mto1+
mto1[NΔ130]
mto1[bonsai]-GFP
C
mCh-Atb2
"Mto1"
merge
mto1-GFP
nmt81:mto1-GFP
mto1[bonsai]-GFP
nmt81:
mto1[bonsai]-GFP
D
mCh-Atb2
"Mto1"
merge
mto1-GFP
mto1[9A1]-GFP
mto1[bonsai]-GFP
mto1[9A1-bonsai]-
GFP
E
Mto1
Mto1[bonsai]
1. MT lattice binding
2. MT nucleation
(and bundling)
1. MT nucleation
2. Diffusion
3. MT bundling
F
Whole Cell Extract
Anti-GFP IP
mto1+
mto1-GFP
mto1[9A1]-GFP
mto1-GFP mto2Δ
mto1[bonsai]
mto1[bonsai]-GFP
mto1[9A1-bonsai]-GFP
mto1[bonsai]-GFP mto2Δ
WB:
Mto1
WB:
Mto2
WB:
Gtb1
G
Co-immunoprecipitated
Gtb1 in anti-GFP IP
Gtb1 integrated intensity
(arbitrary units)
1.0
0.8
0.6
0.4
0.2
0
mto1-GFP
mto1[9A1]-GFP
mto1-GFP mto2Δ
mto1[bonsai]-GFP
mto1[9A1-bonsai]-GFP
mto1[bonsai]-GFP mto2Δ

Supplemental Figure S2: Characterization of Mto1[NΔ130] and Mto1[bonsai].

(A) Localization of Mto1-GFP and Mto1[NΔ130]-GFP, expressed from the endogenous *mto1* promoter, in control cells and after microtubule (MT) depolymerization by methyl benzimidazol-2-yl-carbamate (MBC).

(B) Anti-tubulin immunofluorescence showing time-course and spatial distribution of MT regrowth after cold-induced MT depolymerization in *mto1+* and *mto1[NΔ130]* cells (both with untagged Mto1) and in *mto1[bonsai]-GFP* cells.

(C) Localization of Mto1-GFP and Mto1[bonsai]-GFP, expressed from the endogenous *mto1* promoter or overexpressed from the *nmt81* promoter (~6X overexpression relative to endogenous; [S5]), together with mCherry-tubulin (mCh-Atb2). Overexpressed Mto1-GFP effectively coats the entire MT lattice, while overexpressed Mto1[bonsai]-GFP is seen only in puncta at the minus-ends of recently-nucleated MTs that have become incorporated into MT bundles after nucleation (see also Supplementary Movie S2). Because of the increased fluorescent signal of overexpressed proteins, images of *nmt81:*Mto1-GFP and *nmt81:*Mto1[bonsai]-GFP proteins are scaled approximately four-fold lower (on absolute scale) than images of endogenous expression.

(D) Localization of different versions of Mto1, together with mCherry-tubulin (mCh-Atb2). 1) Mto1-GFP localization to MTs is robust. 2) Mto1[9A1]-GFP localization to MTs results solely from association of the C-terminal region of Mto1 with the MT lattice (see Fig. 1A), because "9A1" proteins cannot interact with the γ-TuSC and do not promote *de novo* cytoplasmic MT nucleation ([S6]; see also Suppl. Fig. S2F,G). As described in main text, the few MTs present in "9A1" mutants are the result of escape of intranuclear mitotic spindle MTs into the cytoplasm [S2]. 3) Mto1[bonsai]-GFP localization to MT bundles results from the fact that Mto1/2[bonsai] remains associated with MT minus-ends after nucleation, as described for panel (C) above and in main text. 4) Mto1[9A1-bonsai]-GFP does not localize significantly to MTs, because it lacks both modes of association with MTs (i.e. lattice-binding and minus-end-binding), and therefore diffuses freely inside cells (see Supplementary Movie S3).

(E) Cartoon summarizing the contrasting mechanisms by which full-length Mto1 and Mto1[bonsai] can become localized to MT bundles *in vivo*.

(F) Anti-Mto1, anti-Mto2, and anti-γ-tubulin (Gtb1) western blots of whole cell extracts and anti-GFP immunoprecipitates from yeast cells expressing the indicated GFP-tagged forms of Mto1, in either wild-type (*mto2+*) or *mto2*Δ backgrounds. Untagged *mto1+* (i.e. wild-type) and *mto1[bonsai]* cells serve as controls.

(G) Quantification of co-immunoprecipitated Gtb1 from anti-GFP immunoprecipitations shown in panel (F). The Gtb1 signal in each immunoprecipitate was divided by the Mto1 signal in the same immunoprecipitate, and these values were then normalized relative to the value from *mto1[bonsai]-GFP* cells. These experiments demonstrate three results: First, Mto1-GFP and Mto1[bonsai]-GFP co-immunoprecipitate similar amounts of γ-tubulin complex and of Mto2. Second, analysis of the "9A1" mutants, in which nine consecutive amino acids within the Mto1 CM1 region are mutated to alanine (see Fig. 1A; [S6]), shows that Mto1[bonsai], like full-length Mto1, requires an intact CM1 region in order to interact with the γ-tubulin complex. Third, analysis of *mto2*Δ mutants shows that Mto1[bonsai], like full-length Mto1, does not interact efficiently with the γ-tubulin complex in *mto2*Δ cells. Bars, 5 μm.

Suppl. Fig. S3

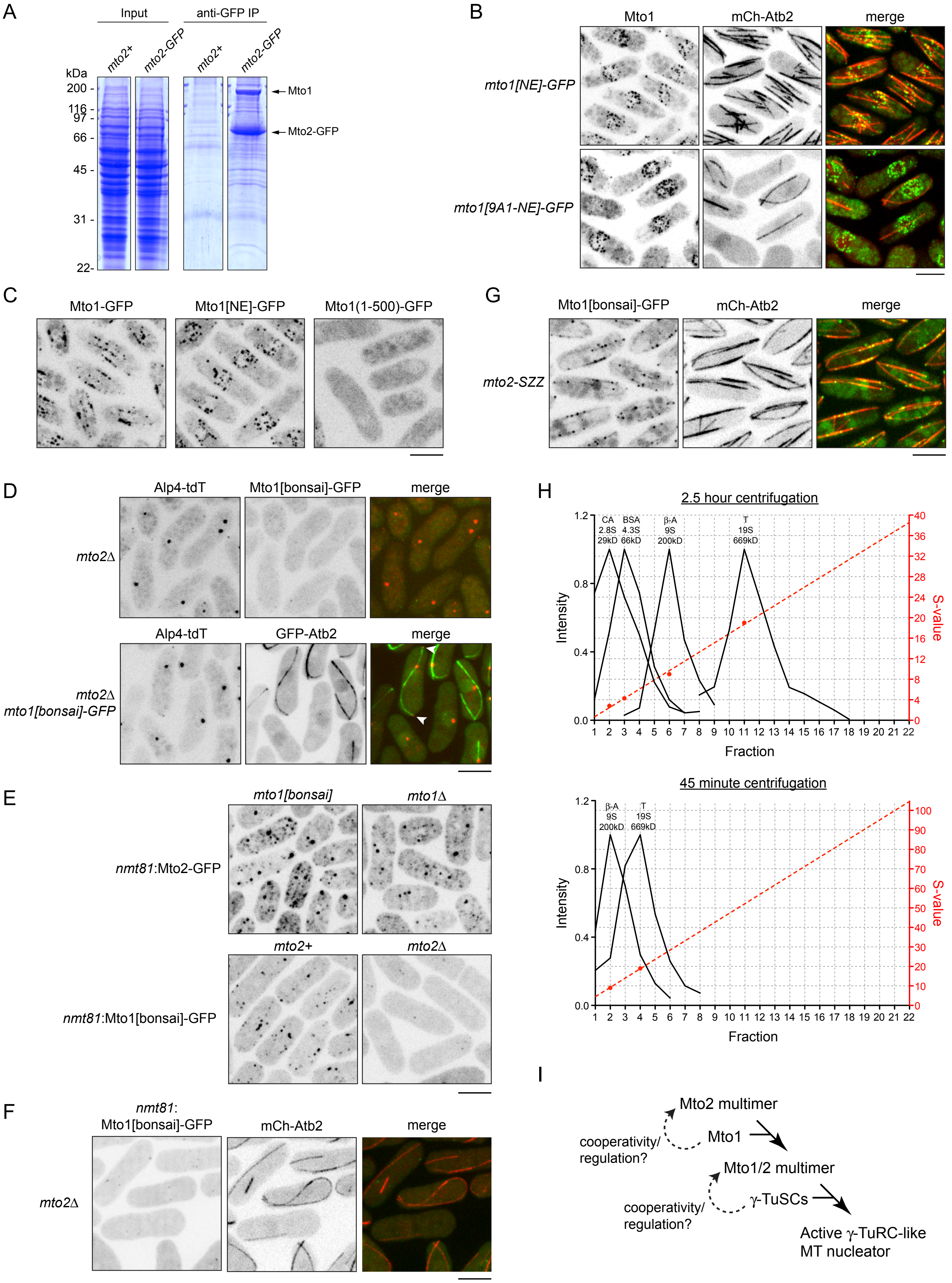
A
Input
anti-GFP IP
mto2+
mto2-GFP
mto2+
mto2-GFP
kDa
200 -
116 -
97 -
66 -
45 -
31 -
22-
Mto1
Mto2-GFP
B
Mto1
mCh-Atb2
merge
mto1[NE]-GFP
mto1[9A1-NE]-GFP
C
Mto1-GFP
Mto1[NE]-GFP
Mto1(1-500)-GFP
G
Mto1[bonsai]-GFP
mCh-Atb2
merge
mto2-SZZ
D
Alp4-tdT
Mto1[bonsai]-GFP
merge
mto2Δ
Alp4-tdT
GFP-Atb2
merge
mto2Δ
mto1[bonsai]-GFP
H
2.5 hour centrifugation
CA 2.8S 29kD
BSA 4.3S 66kD
β-A 9S 200kD
T 19S 669kD
Intensity
S-value
Fraction
45 minute centrifugation
β-A 9S 200kD
T 19S 669kD
Intensity
S-value
Fraction
E
mto1[bonsai]
mto1Δ
nmt81:Mto2-GFP
mto2+
mto2Δ
nmt81:Mto1[bonsai]-GFP
F
nmt81:
Mto1[bonsai]-GFP
mCh-Atb2
merge
mto2Δ
I
Mto2 multimer
cooperativity/
regulation?
Mto1
Mto1/2 multimer
cooperativity/
regulation?
γ-TuSCs
Active γ-TuRC-like
MT nucleator

Supplemental Figure S3: Role of Mto1 and Mto2 in Mto1/2[bonsai] puncta formation.

(A) Coomassie-blue stain of anti-GFP immunoprecipitations from *mto2-GFP* cells and control wild-type (*mto2+*) cells. Mto1 and Mto2 are the only major stoichiometric components. Note that although γ-tubulin complex proteins are readily detected in Mto1/2 immunoprecipitations by Western blot ([S2, S5, S6]; Suppl. Fig. S2F), the majority of Mto1/2-associated γ-tubulin complex does not survive cell extraction and immunoprecipitation. Mto1 rather than Mto1[bonsai] was used in this experiment because untagged Mto1[bonsai] is relatively unstable *in vivo* (see Suppl. Fig. S1F).

(B) Localization of Mto1[NE]-GFP and Mto1[9A1-NE]-GFP puncta to the nuclear envelope, together with mCh-Atb2. Like other "*9A1*" mutants, *mto1[9A1-NE]-GFP* cells are completely defective in cytoplasmic microtubule (MT) nucleation.

(C) Unlike Mto1-GFP and Mto1[NE]-GFP, Mto1(1-500)-GFP is not observed in puncta. This is consistent with Fig. 3A, showing that Mto2 is required for the formation of Mto1-GFP puncta, together with our earlier finding that Mto1 amino acids 461-549 are required for interaction with Mto2 [S5].

(D) Upper panels: Images showing absence of free cytoplasmic Alp4-tdT puncta in *mto2Δ mto1[bonsai]-GFP* cells (compare with Fig. 1G). Lower panels: Images of Alp4-tdT together with GFP-Atb2 in *mto2Δ mto1[bonsai]-GFP* cells. In the absence of Mto1/2 multimers, the γ-TuC does not multimerize into free cytoplasmic puncta, although γ-TuC can still be seen at the SPB, presumably because of association with Pcp1 on the intranuclear face of the SPB (see Suppl. Fig. S1B). Occasionally, very faint Alp4-tdT puncta (white arrows) can be seen at ends of MT bundles, as a result of MT minus-end capping by nucleation-incompetent γ-TuC [S7]. As with *"9A1"* mutants, the few cytoplasmic MTs in these cells result from the escape of intranuclear mitotic spindle MTs into the cytoplasm ([S2]; see also Movie S4).

(E) Images of overexpressed *nmt81:*Mto2-GFP in *mto1[bonsai]* and *mto1Δ* backgrounds, and overexpressed *nmt81:*Mto1[bonsai]-GFP in *mto2*+ and *mto2Δ* backgrounds. *nmt81:*Mto2-GFP forms puncta in the absence of Mto1, while *nmt81:*Mto1[bonsai]-GFP does not form puncta in the absence of Mto2.

(F) Images of *nmt81:*Mto1[bonsai]-GFP together with mCh-Atb2 in an *mto2Δ* background. Even when over-expressed, *nmt81:*Mto1[bonsai]-GFP fails to promote MT nucleation in the absence of Mto2.

(G) Images of Mto1[bonsai]-GFP together with mCh-Atb2 in an *mto2-SZZ* background. This shows that SZZ tagging of Mto2 does not affect MT organization or assembly of Mto1/2[bonsai]-GFP puncta.

(H) Sedimentation profiles of carbonic anhydrase (CA), bovine serum albumin (BSA), β-amylase (β-A), and thyroglobulin (T) size standards on 10-30% isokinetic glycerol gradients, after 2.5-hour and 45-minute centrifugations, as determined by Coomassie-staining of gradient fractions.

(I) Model for assembly of active γ-TuC MT nucleation complexes via Mto1/2-dependent multimerization. In the model, Mto2 multimers provide a fundamental basis for Mto1/2 multimerization, which in turn promotes multimerization of γ-TuSCs. Additional cooperative interactions may "feed back" to regulate more detailed aspects of assembly. Bars, 5 μm.

Supplemental Experimental Procedures

Yeast strain construction and cultures

All strains used in this study are listed below. Standard fission yeast genetic techniques were used throughout [S10]. Truncation, tagging, and deletion of genes were performed using PCR-based methods [S11]. PCR-based methods were used to generate a series of GFP-tagged *mto1* C-terminal truncation mutants: *mto1(1-800)-GFP, mto1(1-732)-GFP, mto1(1-683)-GFP, mto1(1-637)-GFP, mto1(1-575)-GFP, mto1(1-549)-GFP, mto1(1-500)-GFP.* Mutants ranging in size form *mto1(1-549)-GFP* to *mto1(1-800)-GFP* all exhibited the same phenotype, and further analysis was therefore limited to the smallest fragment *mto1(1-549)* (later referred to as *mto1[NE]*). A two-step gene replacement technique was used to generate Mto1 N-terminal truncations expressed from the endogenous *mto1* promoter. In the first step, nucleotides 1-390 of the *mto1* coding sequence were deleted and replaced with the *ura4*+ gene, using PCR-based methods. In the second step, the *ura4*+ gene was replaced with truncated versions of the *mto1* N-terminal coding sequence, produced by overlap-extension ("megaprimer") PCR. The resulting *mto1* N-terminal truncation mutants were selected for resistance to 5-fluoroorotic acid (5-FOA) and confirmed by DNA sequencing. This method was used to create a series of Mto1 N-terminal truncation mutants, where the N-terminus was truncated to amino acid 131, 203, or 242. While Mto1 function was retained following truncation to amino acid 131, further truncation to amino acid 203 or 242 was found to impair Mto1 function: cells had curved morphology, abnormal MT arrays, and rarely nucleated new MTs. As a result, further analysis of Mto1 N-terminal truncations was limited to mutants truncated up to amino acid 131.

S. pombe cells expressing N-terminal 6His-FLAG-GFP-tagged Mto2 under control of the *nmt81* promoter (*nmt81:*HFG-Mto2), were generated by Gateway-based cloning (Life Technologies) followed by homologous recombination at the *leu1* locus. PCR was used to generate the *mto2*+ coding sequence flanked with attB1/2 sites, which was then integrated into the Gateway donor vector pDONR221 using BP Clonase. The *mto2*+ coding sequence was then transferred from pDONR221 into the tagging vector pDUAL-HFG-81c [S12] using LR Clonase. The pDUAL-HFG-81c-*mto2* plasmid was digested with *Not*I restriction enzyme, liberating a *leu1*-targeting fragment containing the *nmt81:HFG-mto2* sequence. This fragment was then used to transform *leu1-32* (leucine-auxotroph) *S. pombe* cells. Upon recombination at the mutant *leu1-32* locus, the *leu1*-targeting fragment confers leucine prototrophy [S12], allowing for selection of Leu+ integrants expressing *nmt81:*HFG-Mto2.

Cells were grown in YE5S rich medium, or in Nurse's modified EMM2 minimal medium [S10] using 5 g/l sodium glutamate instead of ammonium chloride as nitrogen source, and nutritional supplements at 175 mg/l; we refer to this as minimal medium. All cell-imaging experiments used minimal medium, with sodium glutamate and glucose added after autoclaving.

List of strains used in this study:

Strain	Genotype	Source
KS819	*h+ mto1-GFP:kanMX ade6-216 leu1-32 ura4-D18*	Lab stock
KS1017	*h+ mto1Δ::kanMX6 ade6-216 leu1-32 ura4-D18*	Lab stock
KS1407	*h- mto1-GFP:kanMX mto2Δ::kanMX6 ade6-216 leu1-32 ura4-D18*	Lab stock
KS1504	*h- mto2-myc:kanMX ade6-210 leu1-32 ura4-D18*	Lab stock
KS1889	*h+ mto2-GFP:kanMX6 ade6-216 leu1-32 ura4-D18*	Lab stock
KS2076	*h+ mto1(9A1)-GFP:kanMX6 ade6-216 leu1-32 ura4-D18*	Lab stock
KS2738	*h- mto1-GFP:kanMX6 kanMX6:nmt81:GFP-atb2 ade6-216 leu1-32 ura4-D18*	Lab stock
KS2802	*h+ hphMX6:nmt81:GFP-atb2 ade6-210 leu1-32 ura4-D18*	Lab stock
KS3887	*h+ kanMX6:nmt81:mto1-GFP:hphMX ade6-216 leu1-32 ura4-D18*	Lab stock
KS3915	*h- nmt81:His6FLAG:GFP-mto2 mto2Δ:kanMX6 ade6-216 leu1-32 ura4-D18*	This study
KS3920	*h- nmt81:His6FLAG:GFP-mto2 mto2-myc:kanMX6 ade6-210 leu1-32 ura4-D18*	This study
KS515	*h+ ade6-210 leu1-32 ura4-D18*	Lab stock
KS516	*h- ade6-216 leu1-32 ura4-D18*	Lab stock
KS5207	*h- mto1(1-500)-GFP:kanMX6 ade6-210 leu1-32 ura4-D18*	This study
KS5209	*h- mto1(1-549)-GFP:kanMX6 ade6-210 leu1-32 ura4-D18*	This study
KS5349	*h+ mto1(1-549)-GFP:kanMX6 mto2Δ::kanMX6 ade6-216 leu1-32 ura4-D18*	This study
KS5381	*h- natMX6:nmt81:mto1(131-1115)-GFP:kanMX leu1-32 ura4-D18*	This study
KS5385	*h- natMX6:nmt81:mto1(131-549)-GFP:kanMX ade6-210 leu1-32 ura4-D18*	This study
KS5491	*h- mto1(1-549)-GFP:kanMX6 hphMX6:nmt81:GFP-atb2 ade6-210 leu1-32 ura4-D18*	This study
KS5574	*h? nmt81:His6FLAG:GFP-mto2 mto2-myc:kanMX mto1Δ::kanMX6 ade6-216 leu1-32 ura4-D18*	This study
KS5607	*h+ mto1(1-549):kanMX6 ade6-216 leu1-32 ura4-D18*	This study
KS5645	*h- mto1(1-549)-GFP:kanMX6 alp16Δ::natMX6 hphMX6:nmt81:GFP-atb2 ade6-210 leu1-32 ura4-D18*	This study
KS5674	*h- mto1-GFP:kanMX alp4-tdT:natMX6 ade6-216 leu1-32 ura4-D18*	This study
KS5678	*h+ mto1(1-549)-GFP:kanMX6 alp4-tdT:natMX6 ade6-210 leu1-32 ura4-D18*	This study
KS5851	*h- mto1(131-1115) ade6-210 leu1-32 ura4-D18*	This study
KS5922	*h- mto1(131-549)-GFP:kanMX6 ade6-210 leu1-32 ura4-D18*	This study
KS5929	*h+ mto1(131-549)-GFP:kanMX6 hphMX6:nmt81:GFP-atb2 ade6-210 leu1-32 ura4-D18*	This study
KS5933	*h+ mto1(131-549)-GFP:kanMX6 alp4-tdT:natMX6 ade6-210 leu1-32 ura4-D18*	This study
KS5940	*h- mto1(131-1115)-GFP:kanMX6 ade6-210 leu1-32 ura4-D18*	This study
KS5944	*h- mto1(131-1115) hphMX6:nmt81:GFP-atb2 ade6-210 leu1-32 ura4-D18*	This study
KS6080	*h+ mto1(131-9A1-549)-GFP:kanMX6 ade6-216 leu1-32 ura4-D18*	This study
KS6086	*h- mto1(1-549):kanMX6 hphMX6:nmt81:GFP-atb2 ade6-216 leu1-32 ura4-D18*	This study
KS6098	*h- mto1(131-9A1-549)-GFP:kanMX6 mto2Δ::kanMX6 ade6-216 leu1-32 ura4-D18*	This study
KS6102	*h+ mto1(131-1115)-GFP:kanMX6 hphMX6:nmt81:GFP-atb2 ade6-210 leu1-32 ura4-D18*	This study
KS6315	*h+ mto1(131-549)-GFP:kanMX6 alp16Δ::natMX6*	This study

	hphMX6:nmt81:GFP-atb2 ade6-210 leu1-32 ura4-D18	
KS6456	*h- mto1(131-549)-GFP:kanMX6 mto2Δ::kanMX6 ade6 leu1-32 ura4-D18*	This study
KS6457	*h+ mto1(131-549)-GFP:kanMX6 mto2Δ::kanMX6 hphMX6:nmt81:GFP-atb2 ade6 leu1-32 ura4-D18*	This study
KS6458	*h+ mto2Δ::kanMX6 hphMX6:nmt81:GFP-atb2 ade6 leu1-32 ura4-D18*	This study
KS6461	*h+ mto1(1-549)-GFP:kanMX6 mto2Δ::kanMX6 hphMX6:nmt81:GFP-atb2 ade6 leu1-32 ura4-D18*	This study
KS6626	*h- mto1(131-549):kanMX6 ade6-210 leu1-32 ura4-D18*	This study
KS6637	*h? mto1(131-549):kanMX6 hphMX6:nmt81:GFP-atb2 ade6-210 leu1-32 ura4-D18*	This study
KS6672	*h- kanMX6:nmt81:mto1-GFP:hphMX natMX6:Z:ADH15:mCherry-Atb2 ade6 leu1-32 ura4-D18*	This study
KS6673	*h+ mto1-GFP::kanMX natMX6:Z:ADH15:mCherry-Atb2 ade6 leu1-32 ura4-D18*	This study
KS6675	*h+ mto1(1-9A1-549)-GFP:kanMX6 Z:ADH15:mCherry-Atb2:natMX6 ade6 leu1-32 ura4-D18*	This study
KS6676	*h+ mto1(131-9A1-549)-GFP:kanMX6 natMX6:Z:ADH15:mCherry-Atb2 ade6 leu1-32 ura4-D18*	This study
KS6677	*h+ mto1(1-549)-GFP:kanMX6 Z:ADH15:mCherry-Atb2:natMX6 ade6-M210 leu1-32 ura4-D18*	This study
KS6678	*h+ mto1(131-549)-GFP:kanMX6 natMX6:Z:ADH15:mCherry-Atb2 ade6-210 leu1-32 ura4-D18*	This study
KS6680	*h+ natMX6:nmt81:mto1(131-549)-GFP:kanMX natMX6:Z:ADH15:mCherry-Atb2 ade6 leu1-32 ura4-D18*	This study
KS6682	*h- rlc1-GFP:kanMX6 ade6-210 leu1-32 ura4-D18*	This study
KS6715	*h- natMX6:Z:ADH15:mCherry-Atb2 ade6 leu1-32 ura4-D18*	Lab stock
KS6776	*h+ alp4-GFP:hphMX6 mto1(131-549):kanMX6 natMX6:Z:ADH15:mCherry-Atb2 ade6-210 leu1-32 ura4-D18*	This study
KS6791	*h- mto2-GFP:kanMX6 mto1(131-549):kanMX6 natMX6:Z:ADH15:mCherry-Atb2 ade6-210 leu1-32 ura4-D18*	This study
KS6816	*h- mto1-GFP:kanMX alp16Δ::natMX kanMX6:nmt81:GFP-atb2 ade6-216 leu1-32 ura4-D18*	This study
KS6898	*h+ alp6-GFP:kanMX6 mto1(131-549):kanMX6 natMX6:Z:ADH15:mCherry-Atb2 ade6-210 leu1-32 ura4-D18*	This study
KS7055	*h+ mto2-GFP:kanMX6 natMX6:Z:ADH15:mCherry-Atb2 ade6-216 leu1-32 ura4-D18*	This study
KS7056	*h- mto2-GFP:kanMX mto1Δ::kanMX6 natMX6:Z:ADH15:mCherry-Atb2 ade6-216 leu1-32 ura4-D18*	This study
KS7081	*h- mto2-GFP:natMX ade6-210 leu1-32 ura4-D18*	This study
KS7131	*h+ natMX6:nmt81:mto2-GFP:kanMX6 mto1Δ:kanMX ade6-210 leu1-32 ura4-D18*	
KS7134	*h+ mto2-GFP:kanMX6 mto1(1-549):kanMX6 ade6-216 leu1-32 ura4-D18*	This study
KS7160	*h+ mto1(131-549)-GFP:kanMX6 alp4-tdT:natMX6 alp16Δ::natMX6 ade6-M210 leu1-32 ura4-D18*	This study
KS7177	*h+ mto2-SZZ:kanMX6 mto1(131-549)-GFP:kanMX6 Z:ADH15:mCherry-Atb2:natMX6 ade6-M210 leu1-32 ura4-D18*	This study
KS7230	*h- mto1(9A1)-GFP:kanMX6 natMX6:Z:ADH15:mCherry-Atb2 ade6-216 leu1-32 ura4-D18*	This study
KS7319	*h- mto1(131-549)-GFP:kanMX6 mto2Δ:kanMX6 hphMX6:nmt81:GFP-atb2 alp4-tdT:natMX6 ade6-210 leu1-32 ura4-D18*	This study
KS7321	*h- mto1(131-549)-GFP:kanMX6 mto2Δ:kanMX6 alp4-tdT:natMX6 ade6-210 leu1-32 ura4-D18*	This study
KS7367	*h+ mto2-SZZ:kanMX6 mto1Δ::kanMX6 natMX6:Z:ADH15:mCherry-Atb2 ade6-M210 leu1-32 ura4-D18*	This study
KS7384	*h+ mto2-SZZ:kanMX6 mto1(131-9A1-549)-GFP:kanMX6 Z:ADH15:mCherry-Atb2:natMX6 ade6-M210 leu1-32 ura4-D18*	This study

KS7399	*h+ natMX6:nmt81:mto1(131-549)-GFP:kanMX mto2Δ::kanMX6 ade6-210 ura4-D18*	This study
KS7400	*h- natMX6:nmt81:mto2-GFP:kanMX6 mto1(131-549):kanMX6 ade6M216 leu1-32 ura4-D18*	This study

Physiological experiments

To analyze cell morphology of *mto1* C-terminal truncation mutants, cells were grown to stationary phase to disrupt cell polarity, and then allowed to regrow on fresh medium to reveal any polarity phenotypes [S1, S2]. Cells were grown for 2 days on YE5S plates at 32°C, and then replica-plated to fresh YE5S and allowed to grow for 3 hours at 32°C. Cells were then washed from plates using phosphate buffered saline, fixed in 3.7% formalin saline solution for 30 minutes, and imaged by DIC microscopy using a 20X objective.

For microtubule regrowth experiments, cells were grown in YE5S at 30°C to a density of 1×10^7 cells/ml. Microtubules were depolymerized by chilling culture flasks in an ice-water slurry (with occasional mixing) for 30 minutes. Microtubule regrowth was allowed to occur by returning flasks to a water bath at 25°C. Cells were harvested by rapid filtration on 47 mm Durapore membranes (Merck/Millipore) and fixed in -80°C methanol prior to cold-treatment, just before return to warming, and at different times after return to warming. Methanol-fixed cells were processed for immunofluorescence and stained with monoclonal anti-tubulin antibody TAT1 [S13] followed by Alexa568 donkey anti-mouse antibody (Life Technologies). Stained cells were washed and mounted on coverslips [S14] and imaged by spinning-disk fluorescence microscopy (see below).

For imaging Mto1-GFP and Mto1[NΔ130]-GFP localization in the presence of methyl benzimidazol-2-yl-carbamate (MBC*;* also known as Carbendazim), cells were grown in minimal medum. Cells were treated with 25 μg/mL MBC in 1% DMSO prior to imaging by spinning disk confocal microscopy (see below). Minimal medium-agarose pads for imaging (see below) also contained 25 μg/mL MBC and 1% DMSO. Negative control cells were treated with 1% DMSO.

Microscopy and image analysis

Wide-field microscopy was performed using a Nikon TE300 inverted microscope with a Nikon 100x/1.40 NA Plan Apo or Nikon 20X/0.75 NA Plan Apo objective, attached to a Photometrics Coolsnap HQ CCD camera and controlled by Metamorph software (Molecular Devices) [S2]. Spinning-disk confocal microscopy was performed using a Nikon TE2000 inverted microscope with a Nikon 100x/1.45 NA Plan Apo objective, attached to a modified Yokogawa CSU-10 unit (Visitech) and an Andor iXon+ Du888 EMCCD camera, controlled by Metamorph software (Molecular Devices) [S15]. For simultaneous imaging of GFP-tagged Mto1/2 or GFP-tagged γ-TuC proteins with mCherry-tubulin, and also for colocalization imaging of Mto1-GFP and Alp4-tdTomato, an Optosplit III image splitter (Cairn Research Ltd.) with a T560LPXR dichroic filter (Chroma Technology) was used.

Prior to live-cell microscopy, cells were grown in minimal medium at 25°C for two days, with appropriate dilution to maintain exponential growth. Imaging was performed at room temperature (23-25°C). Cells were mounted on thin (50 μm) pads containing minimal medium and 2% agarose, between a coverslip and the microscope slide [S16].

Image processing was performed using Metamorph and ImageJ (NIH) software. Images were adjusted using linear contrast enhancement and are presented as maximum projections containing the full cell volume unless otherwise indicated. Within the same figure panel or movie, all still and time-lapse images were acquired and processed identically and thus can be compared directly, with the exception of overexpressed proteins in Suppl. Fig. S2C (see Suppl. Fig. S2C legend). Movies were generated using Quicktime (Apple) and Fireworks (Adobe).

Conditons for fluorescence microscopy:

**Image type**	**Figures/ Movies**	**Laser power (%)*, exposure time (ms)**		**Z series**	**Timelapse**
		**488 nm**	**561 nm**		
Mto1-GFP, Alp4-tdT**	1G, S1B, S3D	50, 2000	40, 2000	8 x 0.6 um	-
GFP-Atb2	1E, S1E, Movie S1, Movie S4	20, 100	-	9 x 0.5 um	100 x 5 s
*nmt81:*Mto1-GFP +/- MBC	1C	70, 1000	-	9 x 0.6 um	-
Microtubule regrowth	1D, S2B	25, 500	-	11 x 0.4 um	-
Nucleating puncta, mCh-Atb2**	2A, 2B, Movie S2	70, 200	70, 200	8 x 0.6 um	80 x 1.63 s
Mto1-GFP	3A, S3C	70, 500	-	8 x 0.6 um	-
Mto2-GFP, mCh-Atb2**	3B, Movie S5	70, 200	70, 200	8 x 0.6 um	40 x 1.63 s
Mto2-GFP	S1C	70, 500	-	9 x 0.6 um	-
Mto1-GFP +/- MBC	S2A	70, 800	-	8 x 0.6 um	-
Mto1-GFP (endogenous and over-expressed), mCh-Atb2	S2C, S3B	70, 500	40, 500	9 x 0.6 um	-
Mto1-GFP, mCh-Atb2**	S2D	70, 500	70, 500	9 x 0.6 um	-
Mto1[9A1-bonsai]-GFP	Movie S3	70, 500	-	9 x 0.6 um	20 x 4.5 s
nmt81:Mto1[bonsai]-GFP and *nmt81*:Mto2-GFP	S3E	70, 200		9 x 0.6 um	-
nmt81:Mto1[bonsai]-GFP with mCh-Atb2	S3F	70, 500	40, 500	9 x 0.6 um	40 x 5 s
Alp4-tdT, GFP-Atb2 (in *mto1[bonsai]-GFP mto2Δ*)**	S3D	15, 2000	40, 2000	8 x 0.6 um	-
Mto1[bonsai]-GFP, mCh-Atb2 in *mto2-SZZ*	S3G	70, 500	40, 300	8 x 0.6 um	-

* ”100%” power = 20 mW for 488 nm, 15 mW for 561 nm.
** Imaged with Optosplit III image splitter (simultaneous illumination by 488 nm and 561 nm lasers).

For quantification of microtubule nucleation frequency in various *mto1* strains, live-cell movies of GFP-tubulin (*nmt81:*GFP-Atb2) were used, and *de novo* nucleation events were classified as occurring either in the region on/near the nuclear envelope or elsewhere “free” in the cytoplasm (see Fig. 1F and Suppl. Fig. S1G). D*e novo* nucleation events were defined as the appearance of a new, distinct MT that arose neither by breakage of a pre-existing MT nor by release from a pre-existing MT bundle. MT nucleation on pre-existing MTs was explicitly excluded from analysis, because of the difficulties in confidently identifying all such events (which are typically immediately followed by bundling of the new MT with the pre-existing MT on which it was nucleated) and in distinguishing such nucleation events from the sliding of pre-existing MT fragments along other pre-existing MTs. Therefore, for those strains in which the MT-binding region of Mto1 is intact (e.g. wild-type and *mto1[N∆130]*), the true total frequency of MT nucleation is significantly greater than the total nucleation frequency shown in graphs. This should be borne in mind when comparing frequency of “free” vs. “nuclear envelope-associated” MT nucleation among the various strains.

For quantification of fluorescent Mto1[bonsai]-GFP, Mto2-GFP, Alp4-GFP, and Alp6-GFP puncta nucleating mCherry-Atb2-labelled microtubules, images were taken from time-lapse movies using the conditions described for Fig. 2A and Suppl.

Movie S2 (see above). For calibration with Rlc1-GFP, single-timepoint images of an *rlc1-GFP* strain were acquired using identical laser power and exposure time (12 Z-sections; 0.6 μm spacing). All proteins were tagged with the same version of GFP (S65T) to allow for direct comparison of fluorescence intensity per GFP fusion protein. The intensities of nucleating GFP-puncta and Rlc1-GFP cytokinesis nodes were measured in sum projections of two successive Z-sections, using 5x5-pixel circular regions. The average intensity of adjacent cytoplasmic regions (4 regions for Rlc1-GFP and 3 regions for nucleating puncta) was subtracted as background. These cytoplasmic regions were also measured in sum projections of two successive Z-sections, using 5x5-pixel circular regions. The intensity of nucleating puncta was measured at the onset of microtubule nucleation (i.e., at the first timepoint in which nucleation could be detected). To avoid any effects from photobleaching, each preparation was used for one movie only, and the absence of photobleaching was further confirmed by plotting intensity of puncta against the timepoint in the movie at which each punctum was quantified (data not shown); we found no evidence for photobleaching in these experiments. Rlc1-GFP nodes were used as the standard in determining Mto1-bonsai-GFP, Mto2-GFP, Alp4-GFP, and Alp6-GFP copy numbers, using the previously determined average value of 41.3 ± 23 molecules of Rlc1 per cytokinesis node [S17].

Immunoprecipitation experiments

For immunoprecipitations shown in Fig. 3, fission yeast soluble extracts were prepared by freezing cell pellets in liquid nitrogen and then grinding to a powder while frozen. Cell powder was then thawed on ice and resuspended in lysis buffer (50 mM HEPES pH 7.6, 75 mM KCl, 1 mM MgCl2, 1mM EGTA, 0.1% [v/v] Triton X-100, 0.5 mM DTT, 1 mM PMSF, 1 mM benzamidine, 10 μg/ml each of "CLAAPE" protease inhibitors (chymostatin, leupeptin, antipain, aprotinin, pepstatin, E64). Lysates were clarified by centrifugation at 13,000 RPM for 15 minutes at 4°C in a microcentrifuge, and total protein concentration was determined by Bradford assay. Uniform amounts of total protein (~30 mg) were added to ~3x10^{7} Protein G Dynabeads previously bound to 1.2 μg of homemade sheep anti-GFP antibody. Beads were incubated with lysate for 1 hour at 4°C, and then washed six times 1 ml lysis buffer. Protein was eluted from Dynabeads by incubation in Laemmli sample buffer (50°C, 10 min). Western blots of cell extracts ("input") and immunoprecipitates were probed with sheep anti-Mto1 antiserum, sheep anti-GFP antibody, and 9E10 mouse monoclonal anti-Myc antibody, and imaged by enhanced chemiluminescence.

For immunoprecipitations shown in Suppl. Fig. S2, conditions were similar, except lysis buffer was 20 mM NaHEPES pH 7.5, 50 mM K-acetate, 200 mM NaCl, 1 mM EDTA, 0.2% TritonX-100, and a protease inhibitor cocktail: 5 μg/ml CLAAPE, 2 mM AEBSF, 1 mM benzamidine, 1mM PMSF. In addition, Protein G Dynabeads were covalently coupled to homemade sheep anti-GFP antibody using dimethyl pimelimidate. Western blots of cell extracts and immunoprecipitates were probed with sheep anti-Mto1 antiserum, sheep anti-Mto2 antiserum, and GTU-88 monoclonal anti-γ-tubulin antibody (Sigma). Anti-Mto1 and anti-Mto2 blots were further incubated with unlabelled GT-34 mouse monoclonal anti-goat antibody to amplify the signal, and all blots were probed with IRDye800CW donkey anti-mouse antibody. Blots were imaged using an Odyssey fluorescence imager (Licor) and quantified using Image Studio (Licor).

Immunoprecipitations shown in Suppl. Fig. S3 were performed in the same manner as those in Suppl. Fig. S2, except lysis buffer contained 25 mM sodium phosphate pH 7.5, 100 mM KCl, 0.5 mM EDTA, 0.2% Triton X-100, protease inhibitor cocktail (10 μg/mL CLAAPE, 2 mM AEBSF, 2 mM benzamidine, 2 mM PMSF), and phosphatase inhibitors (50 mM Na β-glycerophosphate, 1mM NaF, 0.1 mM Na_3VO_4,

50 nM calyculin A, 50 nM okadaic acid). Cell extracts and immunoprecipitates were analyzed by SDS-PAGE and Coomassie staining.

Quantification of cellular levels of Mto1 and Mto2

To determine absolute concentrations of Mto1 and Mto2 in yeast (i.e. numbers of molecules per cell; Suppl. Fig. S1D), known amounts of recombinant MBP-Mto2 and Mto1-6xHis were used to calibrate Western blots of fission yeast extracts.

The construct for the expression of recombinant Mto1-6xHis was produced by Gateway cloning. PCR was used to amplify the *mto1*+ coding sequence with flanking attB1/2 sites. This was integrated into the Gateway donor vector pDONR201 using BP clonase, and then transferred into the tagging/expression vector p0GWA [S18] (using LR Clonase) for expression of Mto1-6xHis fusion protein. Mto1-6xHis was expressed in BL21-RIL *E. coli*, induced with 1 mM IPTG for 4 hours at 18°C. Cells were lysed by sonication, and fusion protein concentration was determined by SDS-PAGE and Coomassie staining, using bovine serum albumin as a calibration standard.

To produce the construct for expression of MBP-Mto2, PCR was used to amplify the *mto2*+ coding sequence with an N-terminal TEV protease site (not used) and flanking attB1/2 sites. This was integrated into the Gateway donor vector pDONR221 using BP Clonase, and then transferred into the tagging/expression vector pHMGWA [S18], using LR Clonase, for expression of MBP-Mto2 fusion protein. Recombinant MBP-Mto2 was expressed in BL21-CodonPlus-RIL *E. coli* (Stratagene, USA). Protein expression was induced by addition of 0.05 mM IPTG to cultures, which were then grown overnight at 18°C, harvested by centrifugation, and frozen in liquid nitrogen. For purification of MBP-Mto2, 4X pellet volumes of lysis buffer (50 mM Tris-HCl pH 8.0, 150 mM NaCl, 0.2% [v/v] Triton X-100, 1 mM EDTA, 1 mM DTT, 1 mM PMSF, 1 mM benzamidine, 10 µg/ml CLAAPE) were added to frozen cells at 4°C. Cells were resuspended by repeated passage through a Dounce homogenizer (30X loose pestle, 30X tight pestle). Benzonase (2.5 U/ml) and $MgCl_2$ (6mM) were added, and cells were passed 4 times through a French Press pressure cell (ThermoSpectronic, USA) at 15,000 psi. Lysates were clarified by centrifugation at 20,000 RPM in a Beckman JA-20 rotor (20 min, 4°C), followed by a second centrifugation at 40,000 RPM in a Beckman Type 45Ti rotor (1 hour, 4°C). Cleared lysate was incubated with amylose resin (New England Biolabs; 2 ml resin per 20 ml lysate) overnight at 4°C, and resin was then washed with 100 ml of wash buffer (50 mM Tris-HCl pH 8.0, 150 mM NaCl, 0.2% [v/v] Triton X-100, 1 mM EDTA, 1 mM DTT). MBP-Mto2 was then eluted from amylose resin using 10 mM maltose. The concentration of MBP-Mto2 fusion protein was then determined as for Mto1-6xHis (but with correction for the MBP moiety).

To determine the cellular levels of Mto1 and Mto2, total cell extracts were prepared from exponentially growing fission yeast cultures. Pelleted fission yeast cells were resuspended in Tris-buffered saline with 1 mM EDTA and 1 mM PMSF, boiled for 5 minutes to inactivate proteases, and disrupted by bead-beating with 0.5 mm Zirconium beads in a Ribolyser (Hybaid; 2 cycles, 20 s each, speed "6.5"). Disrupted cells were boiled in Laemmli sample buffer (without reducing agents or bromophenol blue), and then centrifuged at 13,000 RPM for 15 minutes at room temperature to pellet insoluble debris. Supernatants were recovered and protein concentration determined by bicinchoninic acid assay (Pierce). Cell extracts corresponding to known amounts of cells were analyzed by quantitative anti-Mto1 or anti-Mto2 western blotting, using an Odyssey fluorescence imager and Image Studio software (Licor). On the same blots, we also analyzed wild-type or *mto1Δ* cell lysates "spiked" with known amounts of recombinant MBP-Mto2 or Mto1-6His, respectively. Lysates spiked with recombinant protein were used to generate a

Western-blot calibration curve, from which the endogenous Mto1 and Mto2 protein levels were calculated.

To determine relative expression levels of Mto1 truncation-mutant proteins compared to full-length Mto1 (Suppl. Fig. S1F), pelleted fission yeast cells were resuspended in Tris-buffered saline, boiled for 5 minutes to inactivate proteases, and disrupted by bead-beating with 0.5 mm Zirconium beads in a Ribolyser (Hybaid), as above. Disrupted cells were boiled in Laemmli sample buffer (without reducing agents or bromphenol blue), and protein concentration of cell extracts was determined by bicinchoninic acid assay (Pierce). Equal amounts of extracts were then analyzed by conventional SDS-PAGE and Western blotting. Western blots were probed with sheep anti-Mto1 antiserum, followed by unlabelled GT-34 mouse monoclonal anti-goat antibody and IRDye800CW donkey anti-mouse antibody. Blots were imaged using an Odyssey fluorescence imager (Licor) and quantified using Image Studio (Licor).

Glycerol gradient analysis of endogenous Mto1/2 and Mto2

Glycerol density-gradient centrifugation of endogenous Mto1/2 and Mto2 (the latter from *mto1*Δ cells) was performed both on cell lysates and on affinity purifications of Mto2 containing a C-terminal SZZ tag. The SZZ tag includes the S-peptide sequence of RNAse A, a TEV cleavage site, and two copies of a minimal synthetic Protein A-derived IgG-binding domain and was generated using the plasmid pKW804 [S19]. Cell pellets were frozen in liquid nitrogen, ground to a powder while frozen, and 40 g of cell powder was resuspended in 80 mL of XB1 lysis buffer (10 mM $NaPO_4$ pH 7.5, 100 mM NaCl, 0.5 mM $MgCl_2$, 1.5 mM EGTA, 1% [v/v] Triton X-100, 5% [v/v] glycerol, 1 mM PMSF, 1 mM AEBSF, 10 µg/ml each of "CLAAPE" protease inhibitors [chymostatin, leupeptin, antipain, aprotinin, pepstatin, E64]). Lysates were clarified by centrifugation at 4000 RPM in a Thermo Scientific Heraeus Megafuge (15 min, 4°C), followed by a second centrifugation at 24,000 RPM in a Beckman JA25.5 rotor (45 min, 4°C). Samples of clarified lysates were analyzed by glycerol density-gradient centrifugation (see below), and the remainder used for Mto2-SZZ purification. For Mto2-SZZ purification, clarified lysates were added to 1.5 mL of IgG-coupled Fractogel beads [S20], which had been previously washed in XB1 buffer. Lysates and beads were allowed to incubate for 2 hours at 4°C. Beads were then washed sequentially with 100 mL of XB1, XB2 (10 mM $NaPO_4$ pH 7.5, 100 mM NaCl, 0.5 mM $MgCl_2$, 1.5 mM EGTA, 0.2% [v/v] Triton X-100, 10% [v/v] glycerol), and XB3 (10 mM $NaPO_4$ pH 7.5, 100 mM NaCl, 0.5 mM $MgCl_2$, 1.5 mM EGTA, 0.1% CHAPS, 10% [v/v] glycerol) buffers. Mto2-S was then released from IgG Fractogel beads by cleavage with 500 units of TEV protease in XB3 buffer (total volume 2.2 mL) for 2 hours at 4°C. Glycerol concentration was reduced to 6% by dilution in XB3 lacking glycerol, and a sample of the TEV-eluted material was concentrated 10-fold by centrifugation in a 30 kDa cut-off centrifugal filter unit (Amicon, Merck/Millipore). Partially purified protein was stored overnight at 4°C prior to glycerol density-gradient centrifugation.

Glycerol gradients (2 mL, 10-30% glycerol [w/v]) were prepared in XB3 buffer using a Gradient Master 107ip (BioComp Instruments). Gradients were loaded with 100 μL of either: clarified lysate; Mto2-S partially purified material; or Mto2-S partially purified and 10-fold concentrated material. Gradients were then centrifuged in a Beckman TLS-55 swinging bucket rotor (55,000 RPM, 4°C) for 2.5 hours or 45 minutes. Size markers were analyzed in parallel on separate gradients. Fractions (100 μL) were collected and analyzed by quantitative anti-Mto1 and anti-Mto2 western blotting (for yeast proteins; see below), or by Coomassie Blue staining (for size markers).

In these experiments, neither the partially purified Mto2-S generated by TEV elution from IgG Fractogel nor associated Mto1[9A1-bonsai]-GFP was observed as a prominent band by SDS-PAGE and Coomassie Blue staining, relative to non-specific

proteins. This is likely due to the very low initial abundance of the proteins, combined with inefficient binding and loss of protein during washes. While the reasons for inefficient purification are not entirely clear, this was not unique to Mto2-SZZ, as we observed considerably worse binding when Mto2 was tagged with other TEV-cleavable tags (not shown). To assess relative concentration and fold-enrichment of Mto1, Mto2, and γ-tubulin following Mto2-S partial purification, we used quantitative Western blotting and quantitative Coomassie Blue staining of partially-purified material versus cell lysates. Mto1 and Mto2 western blots were probed with sheep anti-Mto1 or anti-Mto2 antiserum, followed by unlabelled GT-34 mouse monoclonal anti-goat antibody and IRDye800CW donkey anti-mouse antibody. γ-Tubulin western blots were probed with GTU-88 monoclonal anti-γ-tubulin antibody (Sigma), followed by IRDye800CW donkey anti-mouse antibody. Blots and Coomassie stained gels were imaged using an Odyssey fluorescence imager (Licor) and quantified using Image Studio (Licor). Relative concentration and fold-enrichment were determined as shown for Mto2, and similarly for Mto1[9A1-bonsai]-GFP, with results shown below:

$$\text{Mto2 relative concentration} = \frac{\text{Mto2 - S } \textit{Western blot signal}_{\textit{TEV eluate}}}{\text{Mto2 - SZZ } \textit{Western blot signal}_{\textit{cell lysate}}}$$

$$\text{Mto2 fold - enrichment} = \frac{\left(\dfrac{\text{Mto2 - S } \textit{Western blot signal}_{\textit{TEV eluate}}}{\text{Total } \textit{Coomassie Blue signal}_{\textit{TEV eluate}}}\right)}{\left(\dfrac{\text{Mto2 - SZZ } \textit{Western blot signal}_{\textit{cell lysate}}}{\text{Total } \textit{Coomassie Blue signal}_{\textit{cell lysate}}}\right)}$$

	Cell lysate (counts / μl)	TEV eluate (counts / μl)	Relative concentration in TEV eluate compared to cell lysate	Fold-enrichment as a proportion of total protein
**Partial purification from *mto2-SZZ mto1[9A1-bonsai]-GFP:***				
Total protein	47.3	0.123	0.00260 X	(n/a)
Mto2 (-SZZ or -S)	3.49	30.84	8.84 X	3398 X
Mto1[9A1-bonsai]-GFP	6.46	3.11	0.48 X	185 X
**Partial purification from *mto2-SZZ mto1Δ:***				
Total protein	48.2	0.129	0.00268 X	(n/a)
Mto2 (-SZZ or -S)	4.89	18.53	3.79 X	1417 X

Note: Counts are arbitrary units derived from measurements using the Licor Odyssey. They can be compared within rows but not between rows

In these experiments, γ-tubulin in the TEV eluate was barely detectable, and approximate quantification of γ-tubulin signal was consistent with no enrichment whatsoever. We note that when purified from the *mto1Δ* background, Mto2-S in the TEV eluate was significantly less enriched as a proportion of total protein as compared to when purified from the *mto1[9A1-bonsai]-GFP* background. This may reflect the influence of Mto1 on the multimerization state of Mto2.

Supplemental References

S1. Browning, H., Hayles, J., Mata, J., Aveline, L., Nurse, P., and McIntosh, J.R. (2000). Tea2p is a kinesin-like protein required to generate polarized growth in fission yeast. J Cell Biol *151*, 15-28.

S2. Sawin, K.E., Lourenco, P.C., and Snaith, H.A. (2004). Microtubule nucleation at non-spindle pole body microtubule-organizing centers requires fission yeast centrosomin-related protein mod20p. Curr Biol *14*, 763-775.

S3. Flory, M.R., Morphew, M., Joseph, J.D., Means, A.R., and Davis, T.N. (2002). Pcp1p, an Spc110p-related calmodulin target at the centrosome of the fission yeast Schizosaccharomyces pombe. Cell Growth Differ *13*, 47-58.

S4. Fong, C.S., Sato, M., and Toda, T. (2010). Fission yeast Pcp1 links polo kinase-mediated mitotic entry to gamma-tubulin-dependent spindle formation. EMBO J *29*, 120-130.

S5. Samejima, I., Lourenco, P.C., Snaith, H.A., and Sawin, K.E. (2005). Fission yeast mto2p regulates microtubule nucleation by the centrosomin-related protein mto1p. Mol Biol Cell *16*, 3040-3051.

S6. Samejima, I., Miller, V.J., Groocock, L.M., and Sawin, K.E. (2008). Two distinct regions of Mto1 are required for normal microtubule nucleation and efficient association with the gamma-tubulin complex in vivo. J Cell Sci *121*, 3971-3980.

S7. Anders, A., and Sawin, K.E. (2011). Microtubule stabilization in vivo by nucleation-incompetent gamma-tubulin complex. J Cell Sci *124*, 1207-1213.

S8. Anders, A., Lourenco, P.C., and Sawin, K.E. (2006). Noncore components of the fission yeast gamma-tubulin complex. Mol Biol Cell *17*, 5075-5093.

S9. Zimmerman, S., and Chang, F. (2005). Effects of {gamma}-tubulin complex proteins on microtubule nucleation and catastrophe in fission yeast. Mol Biol Cell *16*, 2719-2733.

S10. Moreno, S., Klar, A., and Nurse, P. (1991). Molecular analysis of the fission yeast *Schizosaccharomyces pombe*. Meth Enzymol *194*, 795-823.

S11. Bahler, J., Wu, J.Q., Longtine, M.S., Shah, N.G., McKenzie, A., 3rd, Steever, A.B., Wach, A., Philippsen, P., and Pringle, J.R. (1998). Heterologous modules for efficient and versatile PCR-based gene targeting in Schizosaccharomyces pombe. Yeast *14*, 943-951.

S12. Matsuyama, A., Shirai, A., Yashiroda, Y., Kamata, A., Horinouchi, S., and Yoshida, M. (2004). pDUAL, a multipurpose, multicopy vector capable of chromosomal integration in fission yeast. Yeast *21*, 1289-1305.

S13. Woods, A., Sherwin, T., Sasse, R., MacRae, T.H., Baines, A.J., and Gull, K. (1989). Definition of individual components within the cytoskeleton of *Trypanosoma brucei* by a library of monoclonal antibodies. J Cell Sci *93*, 491-500.

S14. Sawin, K.E., and Nurse, P. (1998). Regulation of cell polarity by microtubules in fission yeast. J Cell Biol *142*, 457-471.

S15. Snaith, H.A., Anders, A., Samejima, I., and Sawin, K.E. (2010). New and old reagents for fluorescent protein tagging of microtubules in fission yeast; experimental and critical evaluation. Meth Cell Biol *97*, 147-172.

S16. Sawin, K.E. (1999). GFP fusion proteins as probes for cytology in fission yeast. Meth Cell Biol *58*, 123-138.

S17. Laporte, D., Coffman, V.C., Lee, I.J., and Wu, J.Q. (2011). Assembly and architecture of precursor nodes during fission yeast cytokinesis. J Cell Biol *192*, 1005-1021.

S18. Busso, D., Delagoutte-Busso, B., and Moras, D. (2005). Construction of a set Gateway-based destination vectors for high-throughput cloning and expression screening in Escherichia coli. Anal Biochem *343*, 313-321.

S19. Brune, C., Munchel, S.E., Fischer, N., Podtelejnikov, A.V., and Weis, K. (2005). Yeast poly(A)-binding protein Pab1 shuttles between the nucleus and the cytoplasm and functions in mRNA export. RNA *11*, 517-531.

S20. Sawin, K.E., Bicho, C.C., and Snaith, H.A. (2010). Inexpensive synthetic-based matrix for both conventional and rapid purification of protein A- and tandem affinity purification-tagged proteins. Anal Biochem *397*, 241-243.

Movie Legends

Movie S1. *mto1[NE]-GFP* cells exhibit increased microtubule nucleation from the nuclear envelope, while *mto1[bonsai]-GFP* cells exhibit spatially random microtubule nucleation

GFP-tubulin (GFP-Atb2) in *mto1-GFP, mto1[NE]-GFP, and mto1[bonsai]-GFP* cells, in both *alp16+* (wild-type) and *alp16Δ* backgrounds. In *alp16*Δ cells, none of the "γ-TuRC-specific" proteins Gfh1 (GCP4 homolog), Mod21 (GCP5) or Alp16 (GCP6) are associated with the γ-tubulin small complex (γ-TuSC) [S8]. Movies of *alp16+* cells correspond to the images shown in Fig.1E and Suppl. Fig. S1E. GFP-tagged Mto1 is too faint to be seen here relative to GFP-tubulin. All sequences play twice, with asterisks indicating nucleation events during the first run. Z-series were acquired every 5 s. Maximum projections of 9 Z-sections are shown. Movie plays at 15 frames per second (fps).

Movie S2. Microtubules are nucleated directly from puncta containing Mto1[bonsai]-GFP, Mto2-GFP, Alp4-GFP, and Alp6-GFP

mCherry-microtubule (mCh-Atb2) nucleation from Mto1[bonsai]-GFP puncta, and from Mto2-GFP, Alp4-GFP, and Alp6-GFP puncta in an (untagged) *mto1[bonsai]* background. Movies correspond to images shown in Fig. 2A. In the lower Mto1[bonsai]-GFP movie, an Mto1[bonsai]-GFP punctum nucleates an mCherry-microtubule that is subsequently incorporated into a microtubule bundle. Each sequence plays twice, with a box indicating the relevant punctum during the first run. Z-series were acquired every 1.63 s. Movies show single Z-sections or maximum projections of two adjacent Z-sections. Movie plays at 15 fps.

Movie S3. Formation of Mto1 puncta requires Mto2

Mto1[9A1-bonsai]-GFP in *mto2+* (wild-type) and *mto2Δ* cells. Mto1[9A1-bonsai] does not interact with the γ-tubulin complex (Suppl. Fig. S2) and thus allows investigation of Mto1/2 complex organization independently of its association with the γ-tubulin complex. Related examples of other Mto1-variants are shown in Fig. 3A. Z-series were acquired every 4.5 s. Maximum projections of 9 Z-sections are shown. Movie plays at 15 fps.

Movie S4. *mto1[NE]-GFP* and *mto1[bonsai]-GFP* cells are completely defective in cytoplasmic microtubule nucleation in the absence of Mto2

GFP-tubulin (GFP-Atb2) in *mto1+* (wild-type), *mto1[NE]-GFP*, and *mto1[bonsai]-GFP* cells, in both *mto2+* (wild-type) and *mto2Δ* backgrounds. GFP-tagged Mto1 is too faint to be seen here relative to GFP-tubulin. In *mto1+ mto2*Δ cells, cytoplasmic microtubules are nucleated from the spindle pole body only [S5], during both mitosis (astral microtubules from mitotic spindle, right-most cell in bottom-left panel) and interphase (arrow in bottom-left panel). By contrast, in *mto1[NE]-GFP mto2*Δ and *mto1[bonsai]-GFP mto2*Δ cells, cytoplasmic microtubules are never nucleated *de novo* (0 events in 50 cells imaged over 500 seconds; data not shown). Indeed, when cytoplasmic microtubules are present in these cells, they are derived from intranuclear mitotic spindle microtubules that escape into the cytoplasm at the end of mitosis [S2, S6, S9]. If these cytoplasmic microtubules depolymerize completely, they never return, leading to many cells without any cytoplasmic microtubules. Z-series were acquired every 5 s. Max projections of 9 Z-sections are shown. Movie plays at 15 fps.

Movie S5. Formation of Mto2 puncta does not require Mto1

Mto2-GFP and mCherry-tubulin (mCh-Atb2) in *mto1[bonsai]* and *mto1Δ* cells. mCh-Atb2 is also shown in *mto1+ mto2+* cells (wild-type, without any GFP-tagged protein) as a negative control for GFP fluorescence. Movies correspond to images shown in Fig. 3B. When cytoplasmic microtubules are present in *mto1*Δ cells, they are always derived from intranuclear mitotic spindle microtubules that escape into the cytoplasm. Z-series were acquired every 1.63 s for 40 timepoints. Maximum projections of 8 Z-sections are shown. Movie plays at 15 fps.
