# Supplementary figures and images for: Activation of the γ-Tubulin Complex by the Mto1/2 Complex
Source: Curr Biol. 2014 Apr 14;24(8):896–903. doi: 10.1016/j.cub.2014.03.006 (PMC3989768; doi:10.1016/j.cub.2014.03.006)

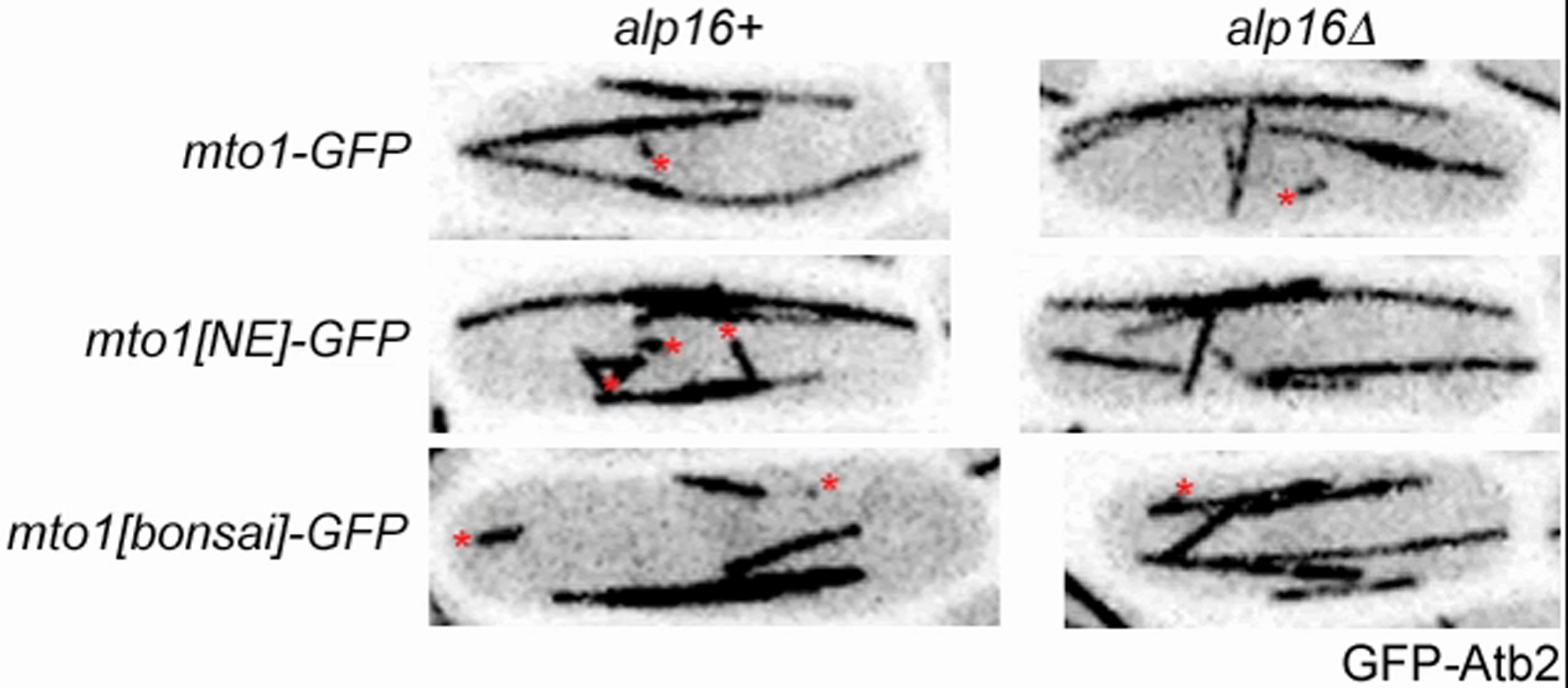

Supplement: Movie S1. mto1[NE]-GFP cells Exhibit Increased Microtubule Nucleation from the Nuclear Envelope, While mto1[bonsai]-GFP Cells Exhibit Spatially Random Microtubule Nucleation — GFP-tubulin (GFP-Atb2) in mto1-GFP, mto1[NE]-GFP, and mto1[bonsai]-GFP cells, in both alp16+ (wild-type) and alp16Δ backgrounds. In alp16Δ cells, none of the “γ-TuRC-specific” proteins Gfh1 (GCP4 homolog), Mod21 (GCP5), or Alp16 (GCP6) are associated with the γ-tubulin small complex (γ-TuSC). Movies of alp16+ cells correspond to the images shown in Figures 1E and S1E. GFP-tagged Mto1 is too faint to be seen here relative to GFP-tubulin. All sequences play twice, with asterisks indicating nucleation events during the first run. z series were acquired every 5 s. Maximum projections of nine z sections are shown. Movie plays at 15 frames per second (fps). [file mmc2.jpg]

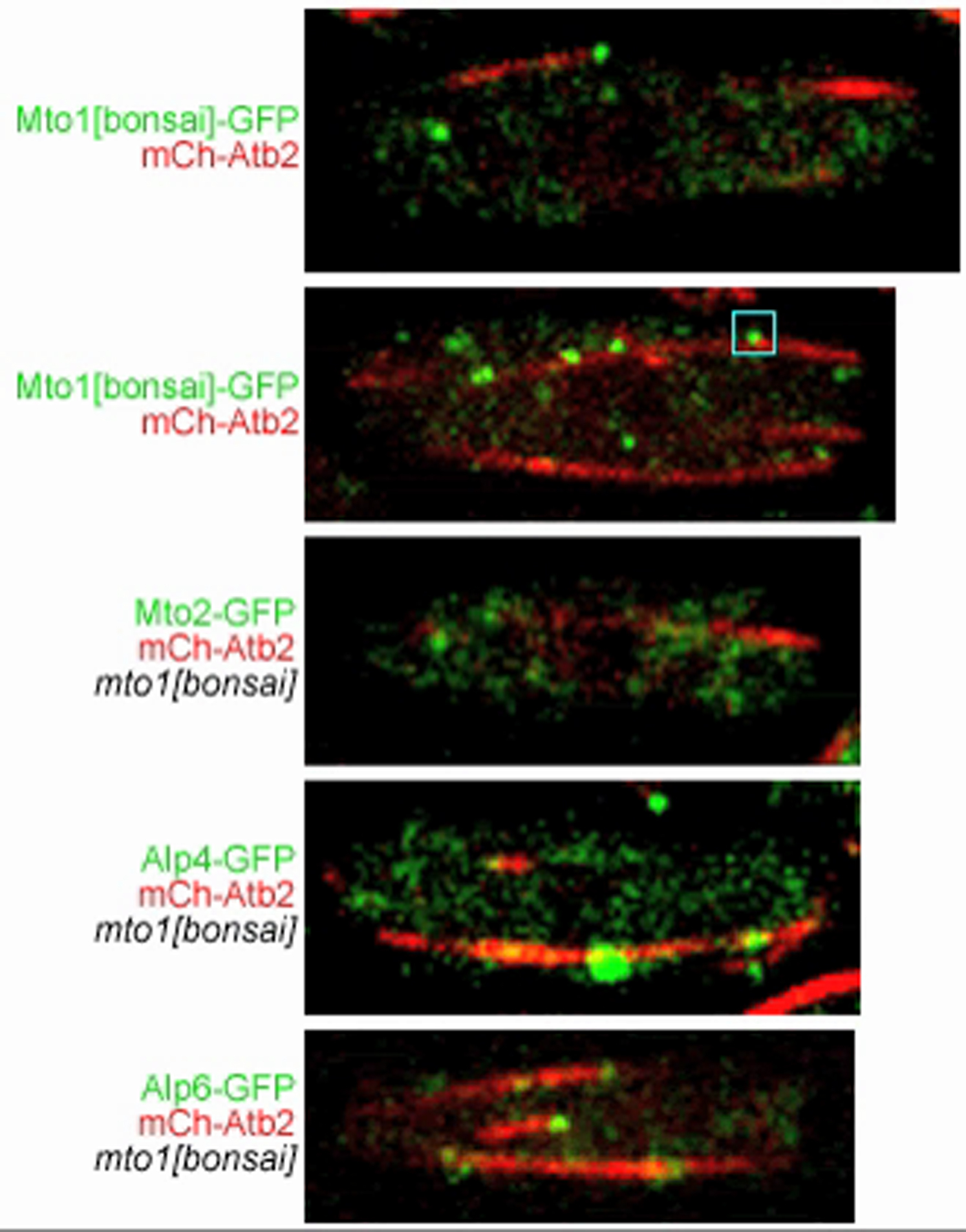

Supplement: Movie S2. Microtubules Are Nucleated Directly from Puncta Containing Mto1[bonsai]-GFP, Mto2-GFP, Alp4-GFP, and Alp6-GFP — mCherry-microtubule (mCh-Atb2) nucleation from Mto1[bonsai]-GFP puncta, and from Mto2-GFP, Alp4-GFP, and Alp6-GFP puncta in an (untagged) mto1[bonsai] background. Movies correspond to images shown in Figure 2A. In the lower Mto1[bonsai]-GFP movie, an Mto1[bonsai]-GFP punctum nucleates an mCherry-microtubule that is subsequently incorporated into a microtubule bundle. Each sequence plays twice, with a box indicating the relevant punctum during the first run. z series were acquired every 1.63 s. Movies show single z sections or maximum projections of two adjacent z sections. Movie plays at 15 fps. [file mmc3.jpg]

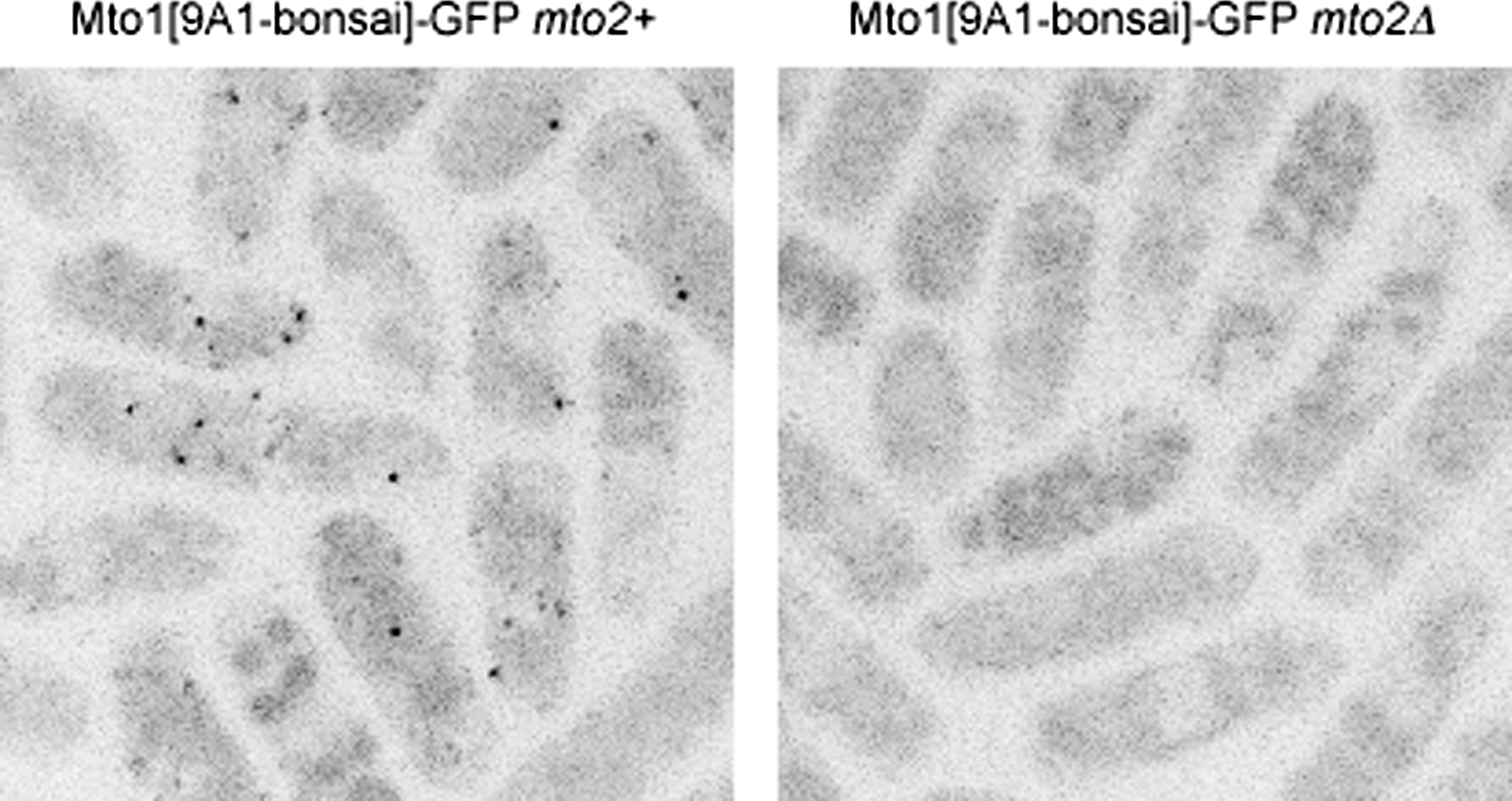

Supplement: Movie S3. Formation of Mto1 Puncta Requires Mto2 — Mto1[9A1-bonsai]-GFP in mto2+ (wild-type) and mto2Δ cells. Mto1[9A1-bonsai] does not interact with the γ-tubulin complex (Figure S2) and thus allows investigation of Mto1/2 complex organization independently of its association with the γ-tubulin complex. Related examples of other Mto1 variants are shown in Figure 3A. z series were acquired every 4.5 s. Maximum projections of nine z sections are shown. Movie plays at 15 fps. [file mmc4.jpg]

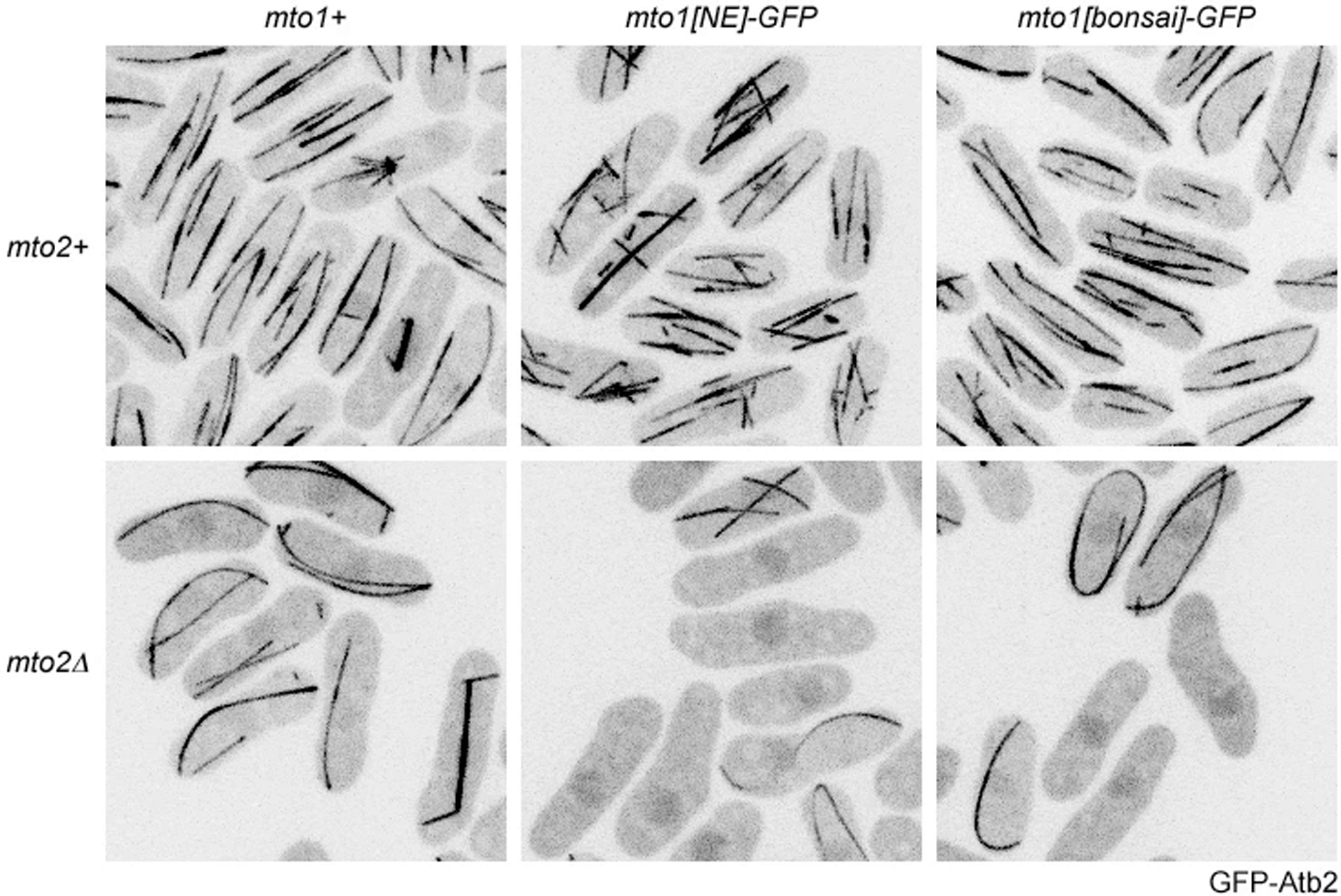

Supplement: Movie S4. mto1[NE]-GFP and mto1[bonsai]-GFP Cells Are Completely Defective in Cytoplasmic Microtubule Nucleation in the Absence of Mto2 — GFP-tubulin (GFP-Atb2) in mto1+ (wild-type), mto1[NE]-GFP, and mto1[bonsai]-GFP cells, in both mto2+ (wild-type) and mto2Δ backgrounds. GFP-tagged Mto1 is too faint to be seen here relative to GFP-tubulin. In mto1+ mto2Δ cells, cytoplasmic microtubules are nucleated from the spindle pole body only, during both mitosis (astral microtubules from mitotic spindle, right-most cell in bottom-left panel) and interphase (arrow in bottom-left panel). By contrast, in mto1[NE]-GFP mto2Δ and mto1[bonsai]-GFP mto2Δ cells, cytoplasmic microtubules are never nucleated de novo (0 events in 50 cells imaged over 500 s; data not shown). Indeed, when cytoplasmic microtubules are present in these cells, they are derived from intranuclear mitotic spindle microtubules that escape into the cytoplasm at the end of mitosis. If these cytoplasmic microtubules depolymerize completely, they never return, leading to many cells without any cytoplasmic microtubules. z series were acquired every 5 s. Max projections of nine z sections are shown. Movie plays at 15 fps. [file mmc5.jpg]

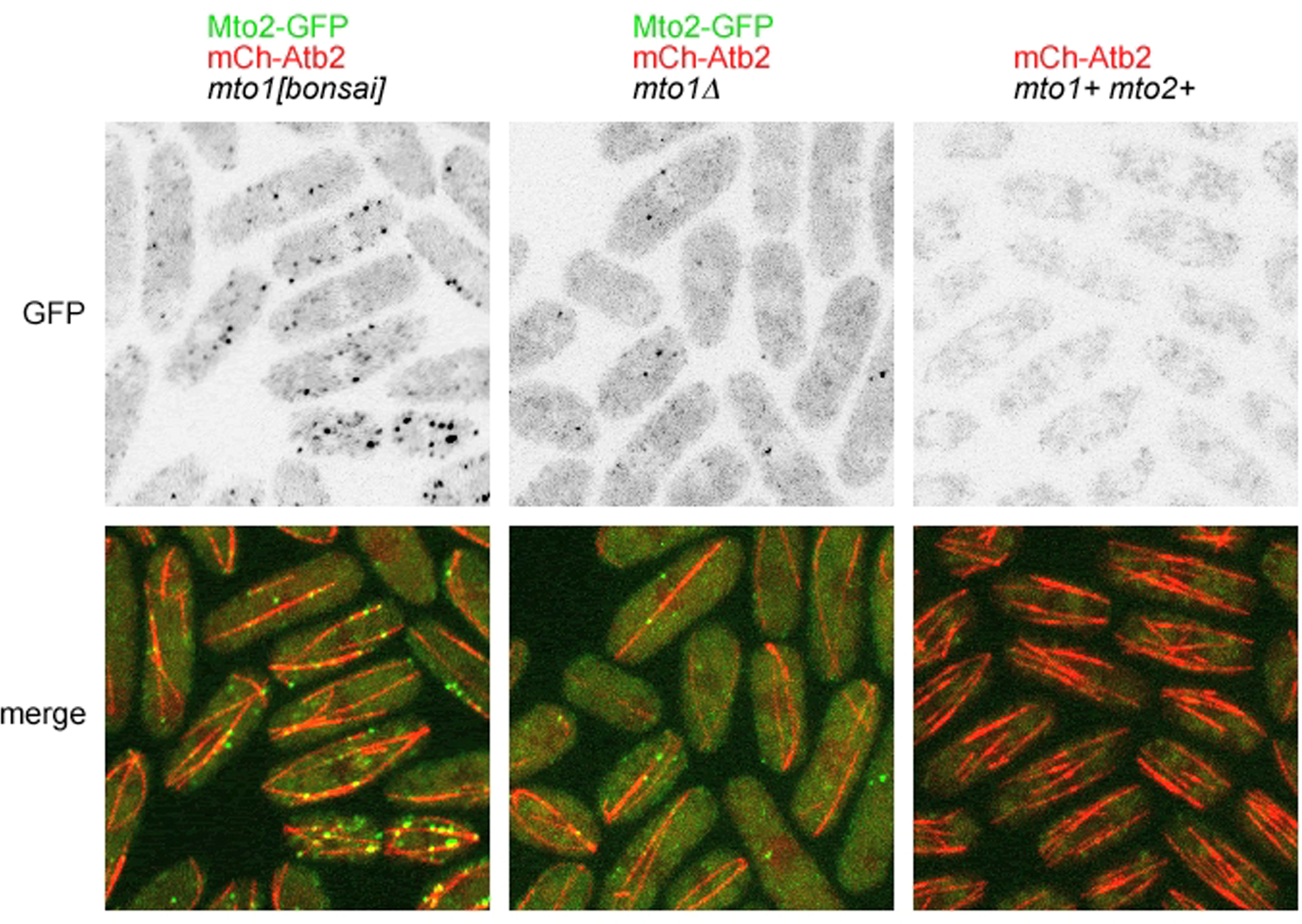

Supplement: Movie S5. Formation of Mto2 Puncta Does Not Require Mto1 — Mto2-GFP and mCherry-tubulin (mCh-Atb2) in mto1[bonsai] and mto1Δ cells. mCh-Atb2 is also shown in mto1+ mto2+ cells (wild-type, without any GFP-tagged protein) as a negative control for GFP fluorescence. Movies correspond to images shown in Figure 3B. When cytoplasmic microtubules are present in mto1Δ cells, they are always derived from intranuclear mitotic spindle microtubules that escape into the cytoplasm. z series were acquired every 1.63 s for 40 time points. Maximum projections of eight z sections are shown. Movie plays at 15 fps. [file mmc6.jpg]
